# Tight junction channel regulation by interclaudin interference

**DOI:** 10.1038/s41467-022-31587-8

**Published:** 2022-06-30

**Authors:** Nitesh Shashikanth, Marion M. France, Ruyue Xiao, Xenia Haest, Heather E. Rizzo, Jose Yeste, Johannes Reiner, Jerrold R. Turner

**Affiliations:** 1 Laboratory of Mucosal Barrier Pathobiology, Department of Pathology, Brigham and Women’s Hospital and Harvard Medical School, Boston, MA USA; 2 Instituto de Microelectrónica de Barcelona, IMB-CNM (CSIC), Bellaterra, Spain; 3 Division of Gastroenterology and Endocrinology, Department of Medicine II, Rostock University Medical Center, Ernst-Heydemann-Str. 6, Rostock, Germany

**Keywords:** Mucosal immunology, Homeostasis, Tight junctions

## Abstract

Tight junctions form selectively permeable seals across the paracellular space. Both barrier function and selective permeability have been attributed to members of the claudin protein family, which can be categorized as pore-forming or barrier-forming. Here, we show that claudin-4, a prototypic barrier-forming claudin, reduces paracellular permeability by a previously unrecognized mechanism. Claudin-4 knockout or overexpression has minimal effects on tight junction permeability in the absence of pore-forming claudins. However, claudin-4 selectively inhibits flux across cation channels formed by claudins 2 or 15. Claudin-4-induced loss of claudin channel function is accompanied by reduced anchoring and subsequent endocytosis of pore-forming claudins. Analyses in nonepithelial cells show that claudin-4, which is incapable of independent polymerization, disrupts polymeric strands and higher order meshworks formed by claudins 2, 7, 15, and 19. This process of interclaudin interference, in which one claudin disrupts higher order structures and channels formed by a different claudin, represents a previously unrecognized mechanism of barrier regulation.

## Abstract (web summary)

Tight junctions are formed by claudin proteins that can be classified as pore- or barrier-forming. Here, Shashikanth et al. report a third function, termed interclaudin interference, in which one claudin inhibits pore function of another claudin by disrupting its polymeric complexes.

## Introduction

Tissue barriers are required for the survival of multicellular organisms. These barriers depend on tight junctions, which seal the paracellular space. Although tight junctions are nearly impermeant in some tissues, selective permeability at many sites, including the kidney and gut, is essential for health. Gastrointestinal tight junctions are cation-selective, i.e., they preferentially allow cation flux. In contrast, distinct segments within the renal tubules are cation- or anion-selective. The magnitude of paracellular ion and water flux and charge-selectivity reflect the specific repertoire of tight junction-associated claudin family proteins expressed.

Claudin proteins have been most simply categorized as pore-forming or barrier-forming. Although charge-selective, the channels created by pore-forming claudins are not as ion-selective as transmembrane ion channels. For example, Cl^−^ is only 10-fold less permeable than Na^+^, and conductances of Li^+^, K^+^, Rb^+^, and Cs^+^ across channels formed by claudin-2, the most well studied pore-forming claudin, are similar to Na^+^^1–4^. These actively gated^5^ channels are also size-selective; they accommodate Na^+^ (1.9 Å diameter) and methylamine (3.78 Å diameter) but largely exclude N-methyl-D-glucamine (7.29 Å diameter).

Knockout (KO) of barrier-forming claudins, e.g., claudin-1, results in death within hours of birth as a consequence of epidermal barrier loss^6^. Conversely, transgenic claudin-4 expression in vitro increases transepithelial electrical resistance (TER) and reduces paracellular cation conductance^7,8^. The manner in which barrier-forming claudins interact with one another as well as pore-forming claudins to limit paracellular flux is unknown, but models developed on the basis of crystal structure data suggest that pore-forming claudin channels may punctuate long polymers composed of barrier-forming claudins^9–12^.

We sought to define how claudin functions are integrated within tight junctions using claudins 2 and 4 as representatives of pore-forming and barrier-forming claudins, respectively. Claudin-4 KO or overexpression had no effect on transepithelial electrical resistance (TER) and caused only small changes in small cation flux across claudin-2-deficient epithelial monolayers. In contrast, when co-expressed with claudins 2 or 15, claudin-4 specifically inhibited channel function. Further structural, morphological, and functional analyses indicate that claudin-4 disrupts higher order claudin structures to inhibit channel function. Because the process involves depolymerization of claudin strands, the foundation on which channels are established, as a fundamental part of this new mechanism, we have termed the overall process *interclaudin interference*. As a whole, these data indicate that claudins must, at a minimum, be categorized not only as pore-forming and barrier-forming but also as regulators of claudin strand structure and pore function.

## Results

### Claudin-4 is not required for epithelial barrier function

**Fig. 1:**
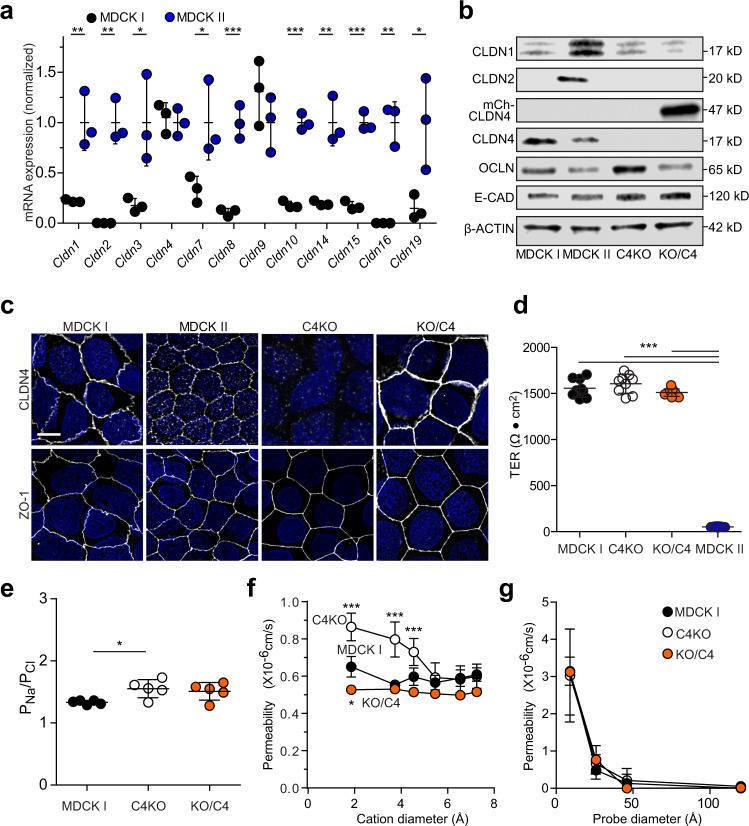
Claudin-4 is not essential for tight junction barrier function. **a** Claudin isoform expression. MDCK I mRNA expression (black symbols) normalized to MDCK II (blue symbols) for each claudin. Some barrier-forming claudins are expressed in both MDCK I and MDCK II, while other barrier-forming and most pore-forming claudins are either absent or expressed at much lower levels in low conductance MDCK I cells. *n* = 3 independent samples. Two-tailed unpaired *t*-test comparisons between MDCK I and MDCK II. P(*Cldn1*) = 0.0082, P(*Cldn2*) = 0.0012, P(*Cldn3*) = 0.0311, P(*Cldn7*) = 0.0435, P(*Cldn8*) = 0. 0008, P(*Cldn10*) < 0.0001, P(*Cldn14*) = 0.0039, P(*Cldn15*) = 0.0002, P(*Cldn16*) = 0.0011, P(*Cldn19*) = 0.035). *P < 0.05; **P < 0.01; ***P < 0.001. **b** Immunoblots of cell lysates from MDCK I, MDCK II, MDCK I claudin-4 KO (C4KO), and C4KO expressing mCherry-claudin-4 (KO/C4). Endogenous claudin-2 (CLDN2), claudin-4 (CLDN4), occludin (OCLN), E-cadherin (E-CAD), β-actin, and transgenically expressed mCherry-claudin-4 (mCh-CLDN4) are shown. Data are representative of 3 independent experiments. Densitometry is shown in Supplementary Fig. 1e. **c** Distributions of claudin-4 and ZO-1 (white) in MDCK I, MDCK II, C4KO, and KO/C4 cells. Nuclei are shown in blue for reference. Maximum projection images are representative of 3 independent experiments. Scale: 10 µm. **d** Peak TERs of MDCK I (black symbols), C4KO (white symbols), and KO/C4 (orange symbols) monolayers are similar to one another and much higher than MDCK II (blue symbols) monolayers. *n* = 8–9, representative of 3 independent experiments. 1-way ANOVA. ***P < 0.0001. **e** Dilution potential measurements show that charge selectivity of paracellular conductance is slightly increased in claudin-4 KO (white symbols) monolayers relative to MDCK I monolayers (black symbols). Charge selectivity of claudin-4 KO monolayers expressing mCherry-claudin-4 (orange symbols) is not significantly different than either MDCK I or claudin-4 KO monolayers. *n* = 5, representative of 3 independent experiments. 1-way ANOVA. *P = .0309. **f** Bi-ionic potential measurements show that claudin-4 KO (white symbols) slightly increases the permeabilities of Na^+^, methylamine, and ethylamine but not larger monovalent cations. Na^+^ permeability of claudin-4 KO monolayers with claudin-4 overexpression (orange symbols) is slightly less than that of MDCK1 monolayers (black symbols). *n* = 4, representative of 3 independent experiments. 1-way ANOVA, *P = 0.018, ***P < 0.0001. **g** Multiplex macromolecular permeability assay using fluorescein and three fluorescent-conjugated dextrans (3 kDa, 10 kDa, 70 kDa) show that neither claudin-4 KO (white symbols) nor overexpression (orange symbols) affects permeability of leak or unrestricted pathways relative to MDCK I monolayers (black symbols). *n* = 8, representative of 3 independent experiments. 1-way ANOVA. Data are presented as mean ± SD and included in the Source Data file.

Previous studies have compared two Madin–Darby Canine kidney lines, MDCK I and MDCK II, with high and low TERs, respectively^13^. Claudin-2 expression in MDCK II, but not MDCK I, is thought to be the key factor that accounts for TER differences between these MDCK clones^14,15^. To explore this further, we surveyed claudin isoform expression and found, as expected, that some barrier-forming claudins, e.g., claudins 4 and 9, were expressed at similar levels in MDCK I and MDCK II cells, but that expression of pore-forming claudins was either reduced or undetectable in MDCK I, relative to MDCK II, cells (Fig. 1a). MDCK II also expressed other claudins classified as barrier-forming that were either absent or expressed at low levels in high TER MDCK I cells (Fig. 1a, b). The repertoire of claudin protein expression in MDCK I and MDCK II cells therefore differs by more than simply the absence of claudin-2 in MDCK I.

To explore the contributions of barrier-forming claudins, we knocked out claudin-4, a prototypic barrier-forming claudin, in MDCK I cells (Fig. 1b, c; Supplementary Fig. 1a). Three independent claudin-4 KO clones displayed modest, statistically insignificant, increases in claudin-1 transcription (Supplementary Fig. 1b). Claudin-1 protein expression was, however, unchanged and both mRNA and protein content remained far less than in MDCK II monolayers (Fig. 1 b, c; Supplementary Fig. 1 b, e). Neither mRNA, protein expression (Fig. 1b and Supplementary Fig. 1c–e), nor distributions of other tight junction proteins (Fig. 1c and Supplementary Fig. 1f) were affected by claudin-4 KO.

Despite conventional wisdom that claudin-4 forms barriers, claudin-4 KO did not reduce TER in high resistance MDCK I monolayers (Fig. 1d), although it did cause a very small, but statistically significant increase in cation selectivity (Fig. 1e) as well as small increases in paracellular permeability of Na^+^, methylamine, and ethylamine (Fig. 1f). Permeability of larger molecules was unaffected by claudin-4 knockout or overexpression (Fig. 1g). Given the classification of claudin-4 as a prototypic barrier-forming claudin, we considered the possibility that, in KO cells, compensation by other claudins allowed maintenance of paracellular barrier function. mCherry-claudin-4 expression in claudin-4 KO MDCK I cells (Fig. 1b, c) caused a minute, though statistically significant, decrease in paracellular Na^+^ methylamine, and ethylamine flux relative to claudin-4 KO (Fig. 1f). mCherry-claudin-4 expression did not, however, affect TER, charge selectivity, or macromolecular permeability (Fig. 1d–g) nor did it alter expression or localization of other tight junction proteins, including claudin-1 (Fig. 1b, c; Supplementary Fig. 1c–f). Claudin-4, therefore, has only limited effects on paracellular Na^+^ conductance and has no significant effect on TER or macromolecular permeability when other barrier-forming claudins are present.

### Claudin-4 reduces permeability by inactivating claudin-2 channels

**Fig. 2:**
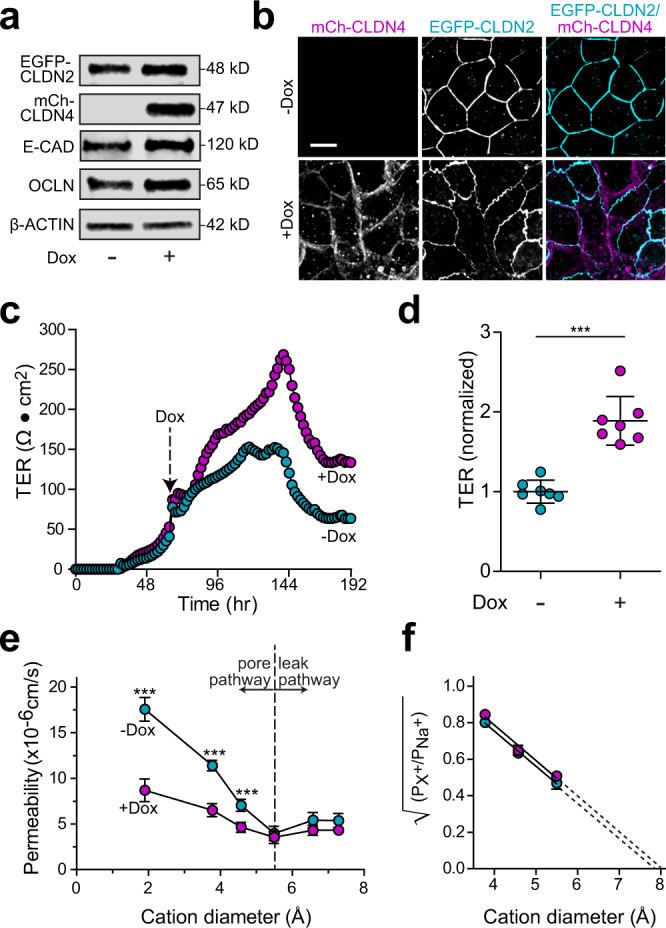
Claudin-4 markedly reduces paracellular small cation conductance in claudin-2 expressing monolayers. **a** Immunoblots of claudin-4 knockout (C4KO) cells expressing EGFP-claudin-2 (EGFP-CLDN2) without (−Dox) or with (+Dox) mCherry-claudin-4 (mCh-CLDN4) expression. Data are representative of 5 independent experiments. **b** EGFP-claudin-2 (cyan in merged image) expressing monolayers without or with doxycycline-induced mCherry-claudin-4 (magenta in merged image) induction. Data are representative of 10 independent experiments. Scale: 10 µm. **c** Continuous TER measurements of EGFP-claudin-2-expressing monolayers (cyan symbols) to which doxycycline was added (magenta symbols) when TER exceeded 50 Ω•cm^2^, after 60 h (arrow) in these traces. Representative of 4 independent experiments. **d** Peak TER of EGFP-claudin-2-expressing monolayers (cyan symbols) is dramatically increased by induction of mCherry-claudin-4 expression (magenta symbols). *n* = 7, representative of 4 independent experiments. Two-tailed unpaired *t*-test. ***P < 0.0001. Results were similar using an independent clone as well as a polyclonal population (Supplementary Fig. 2b). **e** Bi-ionic potential measurements of MDCK I monolayers expressing EGFP-claudin-2 (−Dox, cyan symbols) show that mCherry-claudin-4 expression (+Dox, magenta symbols) specifically reduces permeabilities of Na^+^, methylamine, and ethylamine but not larger cations. *n* = 12, representative of 3 independent experiments. Two-tailed unpaired *t*-test. ***P < 0.0001. **f** Renkin plot showing the square root of permeability ratios of larger monovalent cations relative to Na^+^ as a function of cation diameter. Linear regression shows that the x-intercept, which indicates claudin-2 channel diameter, is identical in monolayers without (cyan symbols) and with (magenta symbols) mCherry-claudin-4 expression. The calculated x-intercept, which is an estimate of claudin-2 pore diameter, is 7.9 Å and 8.0 Å, for EGFP-claudin-2 pores, without and with mCherry-claudin-4 expression, respectively. *n* = 8, representative of 3 independent experiments. Data are presented as mean ± SD and included in the Source Data file.

The failure of mCherry-claudin-4 overexpression to enhance TER in MDCK I monolayers conflicts with a previous report showing that inducible expression of untagged claudin-4 increased TER of MDCK II cell monolayers^7^. Although it is possible that mCherry-claudin-4 was nonfunctional, similarly tagged claudins behave normally in vitro and in vivo^16–19^. Alternatively, the use of MDCK II monolayers in the previous study could explain the discordant results. To model MDCK II monolayers^1,14^, we expressed EGFP-claudin-2 constitutively and mCherry-claudin-4 inducibly in claudin-4 KO MDCK I cells (Fig. 2a, b). EGFP-claudin-2-expressing claudin-4 KO cells were initially plated without doxycycline. Once TER exceeded 50 Ω•cm^2^, indicating development of a confluent monolayer, doxycycline was added to induce mCherry-claudin-4 expression. This caused TER to increase sharply relative to non-induced monolayers (Fig. 2c). Both steady-state and peak TERs of EGFP-claudin-2-expressing monolayers were nearly doubled after mCherry-claudin-4 expression (Fig. 2c, d), similar to the previous study using MDCK II monolayers^7^. These data show that mCherry-claudin-4 is functional and, more importantly, that claudin-2 expression is a prerequisite for claudin-4-induced TER elevation. Consistent with loss of channel function but not wholesale channel modification, the pore size of claudin-2 channels was unaffected by claudin-4 expression (Fig. 2e, f). Claudin-4 may therefore specifically inhibit claudin-2 channels.

### Claudin-4 reduces claudin-2 anchoring at tight junctions

**Fig. 3:**
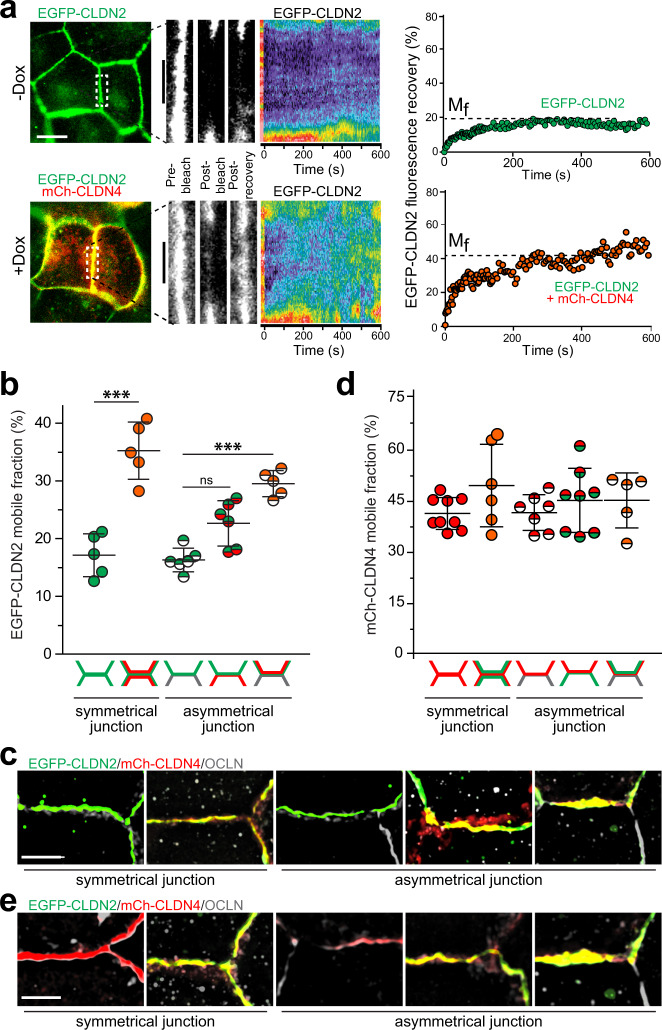
Claudin-4-destabilizes claudin-2 via *cis*-interactions. **a** FRAP analysis of EGFP-claudin-2 at bicellular tight junction. Representative low magnification images show EGFP-claudin-2 (green) and mCherry-claudin-4 (red) before bleaching. High magnification images of the boxed area before and after bleaching. Kymographs (pseudocolor) and quantitative analyses show EGFP-claudin-2 recovery at the tight junctions in cells without (green symbols) and with (orange symbols) mCherry-claudin-4 expression. Representative of at least *n* = 6 in each of 3 independent experiments. Scale: 10 µm, 5 µm. **b** EGFP-claudin-2 FRAP at symmetrical and asymmetrical bicellular junctions. The diagrams beneath each data set indicate which cell(s) express EGFP-claudin-2 (green) or mCherry-claudin-4 (red), i.e., the left column shows a symmetrical bicellular junction at which both cells express EGFP-claudin-2, the second column shows a symmetrical bicellular junction at which both cells express EGFP-claudin-2 and mCherry-claudin-4, and the middle column shows an asymmetrical bicellular junction at which one cell expresses EGFP-claudin-2 and the other expresses no fluorescent claudins (gray). The EGFP-claudin-2 mobile fraction is increased when both proteins are expressed in the same cell (orange symbols) but not when EGFP-claudin-2 (green symbols) and mCherry-claudin-4 (red symbols) are expressed in adjacent cells. *n* = 5–6, representative of 3 independent experiments. 1-way ANOVA ***P < 0.001, *P* = 0.0004. **c** Maximum projection micrographs corresponding to the 5 conditions in which EGFP-claudin-2 (green) FRAP was assessed. mCherry-claudin-4 (red) and occludin (gray) are also shown. Scale: 10 µm. **d** mCherry-claudin-4 FRAP at symmetrical and asymmetrical junctions. Diagrams and graph symbols are colored as in panel **b**. EGFP-claudin-2 expression has no effect on mCherry-claudin-4 mobile fraction. *n* = 5–9, representative of 3 independent experiments. 1-way ANOVA. **e** Micrographs corresponding to the 5 conditions in which mCherry-claudin-4 (red) FRAP was assessed. EGFP-claudin-2 (green) and occludin (gray) are also shown. Scale: 10 µm. Data are presented as mean ± SD and included in the Source Data file.

Tight junction protein complexes undergo continuous molecular remodeling, even at steady-state^20^. This has functional consequences, as reduced claudin-2 anchoring at tight junctions correlates with reduced channel function in vitro and in vivo^16,19^. We therefore hypothesized that claudin-4 might mobilize claudin-2 at tight junctions. Fluorescence recovery after photobleaching (FRAP) analysis confirmed that claudin-2 is largely anchored at the tight junction^19^, with a mobile fraction of 16 ± 3% (Fig. 3a, b). mCherry-claudin-4 expression doubled the EGFP-claudin-2 mobile fraction to 32 ± 4% (Fig. 3a, b). Claudin-4 therefore reduces claudin-2 anchoring at tight junctions.

Interclaudin interactions in both *cis* (in the same cell) and *trans* (in adjacent cells) are required for claudin polymerization^11,21^. To determine which interactions direct anchoring and claudin-4-induced claudin-2 mobilization, mixtures of claudin-4 KO MDCK I cells expressing no transgenes, EGFP-claudin-2, mCherry-claudin-4, or both EGFP-claudin-2 and mCherry-claudin-4 were plated (Fig. 3b). The claudin-2 mobile fraction at asymmetric junctions where EGFP-claudin-2 was expressed in only one cell was 19 ± 2%, similar to that of EGFP-claudin-2 at symmetric junctions (Fig. 3b). Homotypic *trans* interactions are therefore unnecessary for claudin-2 anchoring. Moreover, claudin-4-mediated claudin-2 mobilization does not involve *trans* interactions, as expression of EGFP-claudin-2 and mCherry-claudin-4 on opposite sides of asymmetric junctions did not affect claudin-2 anchoring (Fig. 3b). In contrast, the claudin-2 mobile fraction nearly doubled, to 29 ± 1%, when EGFP-claudin-2 and mCherry-claudin-4 were expressed in *cis* (Fig. 3b). Claudin-4 therefore mobilizes claudin-2 via *cis*, but not *trans*, interactions (Fig. 3b).

The data above suggest that stable binding interactions between claudins 2 and 4 might underlie claudin-4-mediated claudin-2 mobilization. Such binding to claudin-2 could, potentially, affect anchoring of claudin-4, which has a much greater mobile fraction than claudin-2^19,22^. However, the claudin-4 mobile fraction was unchanged (Fig. 3d). Thus, although claudin-4 mobilizes claudin-2, claudin-2 does not affect claudin-4 anchoring at tight junctions.

### Claudin-15 anchoring and channel function are disrupted by claudin-4

**Fig. 4:**
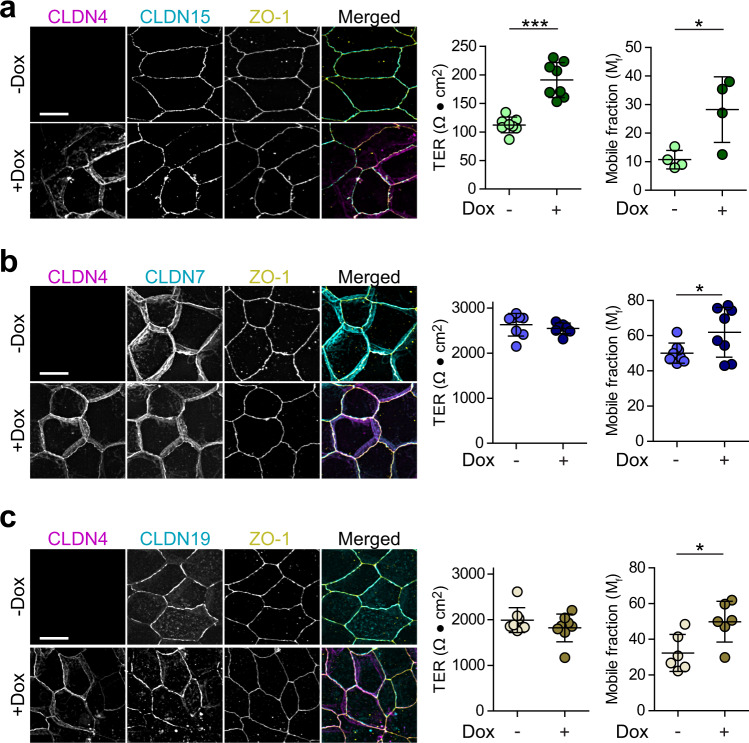
Claudin-4 reduces paracellular conductance across claudin-15, but not claudin-7 or claudin-19, channels. **a** Maximum projection images of MDCK I C4KO monolayers with constitutive expression of EGFP-claudin-15 (cyan in merged image) without (−Dox, light green symbols) or with (+Dox, dark green symbols) mCherry-claudin-4 expression. ZO-1 immunostaining is shown for reference. Merged images show claudin-15 (cyan), claudin-4 (magenta) and ZO-1 (yellow). TER of monolayers expressing EGFP-claudin-15 is low, similar to EGFP-claudin-2-expressing monolayers and is markedly increased by expression of mCherry-claudin-4 (+Dox). FRAP analysis shows that the mobile fraction of EGFP-claudin-15 is increased by mCherry-claudin-4 expression. *n* = 8 (TER) and 8 (FRAP), respectively, representative of 3 independent experiments. Two-tail unpaired *t*-test. *P = 0.021; ***P < 0.0001. Scale: 10 µm. **b** Maximum projection images, TER, and FRAP analysis of MDCK I C4KO monolayers with constitutive expression of EGFP-claudin-7 without (−Dox, light blue symbols) or with (+Dox, dark blue symbols) mCherry-claudin-4 expression. Merged images show claudin-7 (cyan), claudin-4 (magenta) and ZO-1 (yellow). Claudin-4 expression does not change TER of claudin-7-expressing cells but increases the claudin-7 mobile fraction. *n* = 8 (TER) and 8 (FRAP), respectively, representative of 3 independent experiments. Two-tail unpaired *t*-test. *P = 0.047 (FRAP). Scale: 10 µm. **c** Maximum projection images, TER, and FRAP analyses of MDCK I C4KO monolayers with constitutive EGFP-claudin-19 expression without (−Dox, light brown symbols) or with (+Dox, dark brown symbols) mCherry-claudin-4 expression. Merged images show claudin-19 (cyan), claudin-4 (magenta) and ZO-1 (yellow). Scale: 10 µm. Claudin-4 does not affect TER of claudin-19-expressing cells but does induce a small increase in the claudin-19 mobile fraction. *n* = 8 (TER) and 6 (FRAP), respectively, representative of 3 independent experiments. Two-tail unpaired *t*-test. *P = 0.019 (FRAP). Scale: 10 µm. Data are presented as mean ± SD and included in the Source Data file.

Our unexpected results indicate that claudin-4 increases barrier function of claudin-2-expressing monolayers by mobilizing claudin-2 and blocking channel activity. We next asked if claudin-4 could also regulate claudins 7, 15, or 19, which have been classified as pore-forming.^23–27^ When expressed in claudin-4 KO MDCK I, claudin-15 markedly reduced TER (Fig. 4a), similar to the effect of expressing claudin-2. FRAP analysis showed that, also like claudin-2, claudin-15 at tight junctions was largely immobile (Fig. 4a). Claudin-4 expression increased both TER and claudin-15 mobile fraction (Fig. 4a). The effects of claudin-4 on claudin-15 anchoring and channel function are, therefore, comparable to those of claudin-4 on claudin-2. In contrast to claudins 2 and 15, neither claudin-7 (Fig. 4b) nor claudin-19 (Fig. 4c) expression affected TER (Fig. 4b, c). Claudin-4 did, however, cause modest increases in claudin-7 (Fig. 4b) and claudin-19 (Fig. 4c) mobile fractions. Thus, claudin-4 regulates anchoring and function of claudin-15, and anchoring of claudins 7 and 19, in a manner similar to claudin-2.

### Claudin-2 polymers are disrupted by claudin-4

**Fig. 5:**
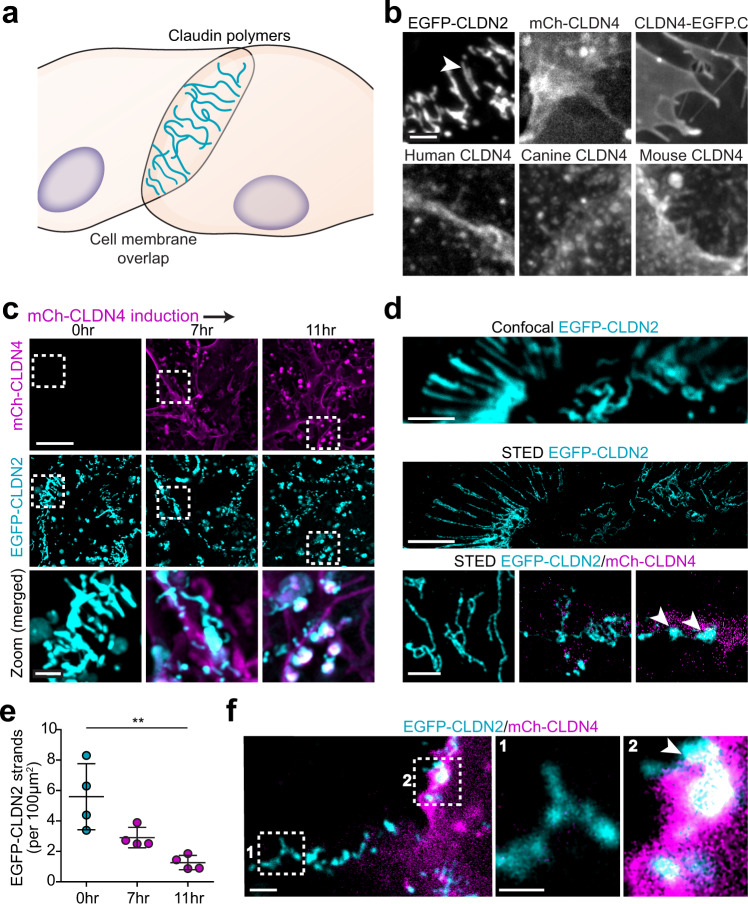
Claudin-4 fails to assemble into polymeric strands but disrupts those formed by claudin-2. **a** Claudin proteins expressed in nonepithelial cells form polymers similar to those within epithelial tight junction strands. **b** EGFP-claudin-2 expressed in U2OS human osteosarcoma cells forms strands (arrowhead). In contrast, mCherry-claudin-4 (human), claudin-4-EGFP (CLDN4-EGFP.C), untagged human claudin-4, canine claudin-4, and mouse claudin-4 all fail to form strands despite being trafficked to plasma membranes. Maximum projection images are representative of 3 independent experiments. Scale: 2 μm. **c** Live imaging at intervals after induction of mCherry-claudin-4 (magenta) expression shows progressive disruption of EGFP-claudin-2 (cyan) strands. Maximum projection images representative of 5 independent experiments. Scale: 10 μm; 2 μm. **d** Confocal and corresponding STED image of EGFP-claudin-2 polymers (cyan) without claudin-4 expression. STED shows collapse of EGFP-claudin-2 (cyan) polymers into dense aggregates (arrowhead) following mCherry-claudin-4 (magenta) expression (bottom). Representative of at least 7 independent experiments. Scale: 1 µm. **e** Quantitative morphometry shows progressive loss of EGFP-claudin-2 strands as mCherry-claudin-4 expression increases. *n* = 4 (each point is the mean of 12–16 cells in 1 sample), representative of 6 independent experiments. 1-way ANOVA. **P = 0.0035. **f** EGFP-claudin-2 strands (box 1) are replaced by amorphous aggregates at sites of asymmetric mCherry-claudin-4 expression (box 2), suggesting that *cis* interactions are sufficient for claudin-4-mediated disruption of claudin-2 strands. Maximum projection images representative of 8 independent experiments. Scale: 1 µm. Data are presented as mean ± SD and included in the Source Data file.

To determine the mechanisms by which claudin-4 mobilizes claudin-2, we asked if claudin-4 modifies the structure of tight junction strands formed by claudin-2. Although tight junction strands cannot be detected using available light microscopic approaches, claudin expression in undifferentiated, nonepithelial cells leads to assembly of strand-like polymers at sites of cell-cell overlap (Fig. 5a). EGFP-claudin-2 expressed in nonepithelial cells readily formed strands but, unexpectedly, mCherry-claudin-4 did not (Fig. 5b). This was not due to the mCherry tag, as strands were not present in nonepithelial cells expressing untagged human claudin-4 or claudin-4-EGFP, nor did it reflect species variation, as neither untagged mouse nor untagged canine claudin-4 formed strands (Fig. 5b). Thus, in contrast to all claudins previously studied in similar reductionist systems^11,28–31^, claudin-4 does not form polymeric strands.

Induction of mCherry-claudin-4 expression after EGFP-claudin-2 strand assembly led to progressive fragmentation, collapse, and dissolution (Fig. 5c, d). By 11 h after doxycycline addition, when mCherry-claudin-4 expression had achieved maximum levels, claudin-2 strand numbers were drastically reduced (Fig. 5e) and largely replaced by amorphous aggregates (Fig. 5d, arrowheads). Aggregates were also present at interfaces where both cells expressed EGFP-claudin-2 but only one expressed mCherry-claudin-4 (Fig. 5f, arrowhead), suggesting that, similar to epithelial tight junctions, *cis* interactions are sufficient for the effects of claudin-4 on claudin-2 polymers.

### Claudin-4 has distinct effects on claudin 7, 15, and 19 strand networks

**Fig. 6:**
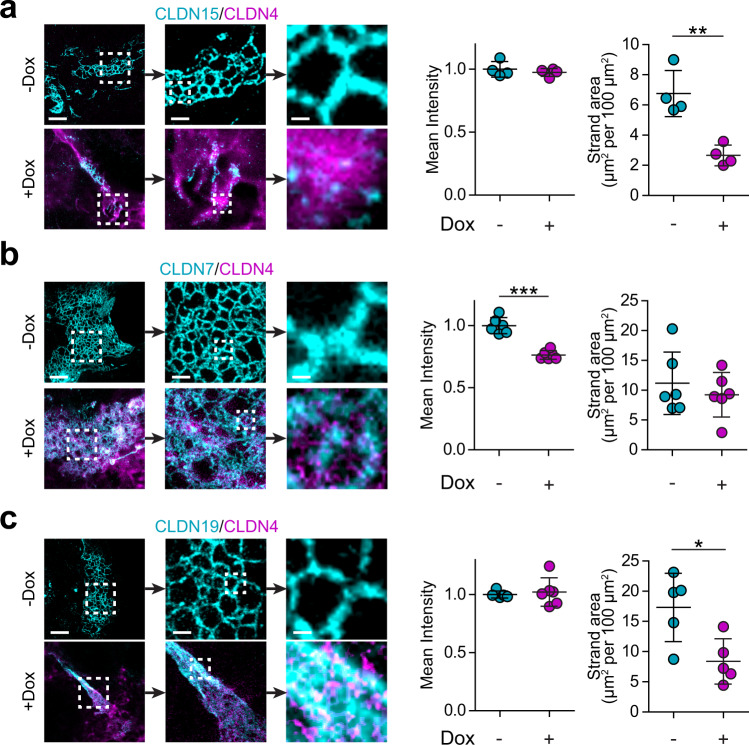
Claudin-7, 15, and 19 strands are modified by claudin-4 expression. **a** STED images show claudin-15 (cyan) meshworks are depolymerized by claudin-4 (magenta), similar to claudin-2 (see Fig. 5). *n* = 4 each for intensity and area, representative of 3 independent experiments. Two-tail unpaired *t*-test. **P = 0.003. **b** Claudin-7 (cyan) strand intensity is reduced and meshworks are rearranged in response to claudin-4 (magenta) expression. *n* = 4 each for intensity and area, representative of 3 independent experiments. Two-tail unpaired *t*-test. ***P < 0.0001. **c** Claudin-19 (cyan) strand meshworks collapse and form dense bundles upon claudin-4 (magenta) expression. *n* = 3 each for intensity and area, representative of 3 independent experiments. Two-tail unpaired *t*-test. *P = 0.019 (area). For each image set, progressive zooms are shown from left to right. Scales: 2 µm, 500 nm, 100 nm. In all plots, each data point represents the mean of 12–16 cells in 1 sample. Data are presented as mean ± SD and included in the Source Data file.

The observations related to claudin-2 strand-like polymers prompted us to ask if claudin-4 had similar effects on claudins 7, 15, and 19. When expressed in nonepithelial cells, all three of these claudins formed strands (Fig. 6a–c). Induction of claudin-4 expression disrupted the claudin-15 meshwork and greatly reduced both the number of strands and the area occupied by strand networks but did not diminish the fluorescent intensity of remaining strands (Fig. 6a). In contrast, mCherry-claudin-4 expression caused the network formed by EGFP-claudin-7 to rearrange and reduced spaces within the mesh (Fig. 6b). Although the area occupied by the meshwork did not change, the mean intensity was reduced by 30% (Fig. 6b). Finally, mCherry-claudin-4 expression caused the network formed by EGFP-claudin-19 to collapse into thick bundles (Fig. 6c). As a result, the area occupied by EGFP-claudin-19 structures was reduced but the fluorescent intensity was unchanged (Fig. 6c). Claudin-4, therefore, modulates strand and meshwork structures formed by claudins 2, 7, 15, and 19 distinctively. The effects on claudins 2 and 15 are nearly identical, consistent with claudin-4 effects on mobile fraction and TER of cells expressing these cation channel-forming claudins. In contrast, claudin-4 had no effect on TER of monolayers expressing claudins 7 or 19, only modestly increased tight junction-associated mobile fractions, and had different effects on the strand meshworks formed by these claudins. Finally, mCherry-claudin-4 overlapped with, but did not integrate into, claudin 2, 7, 15, or 19 polymers.

### Claudin-2 strand disruption is followed by endocytosis

**Fig. 7:**
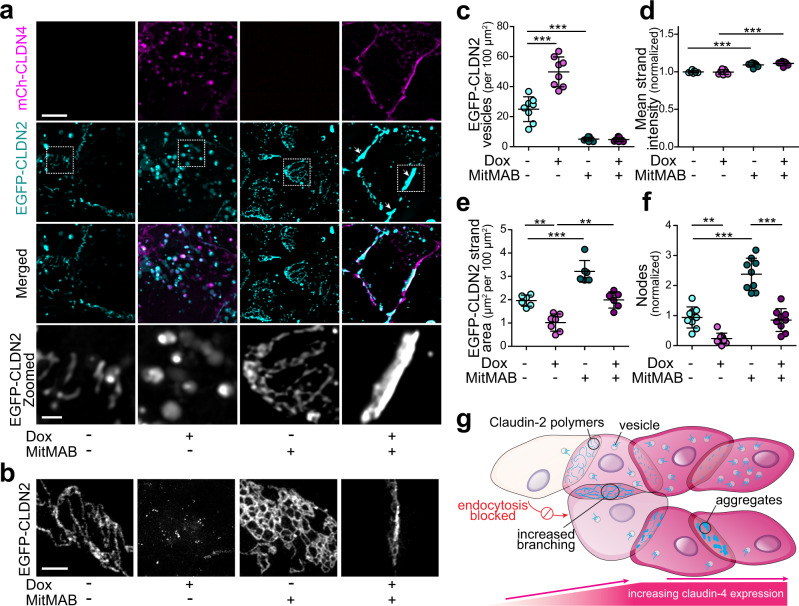
Endocytic blockade is insufficient to preserve claudin-2 strand structure. **a** Dynamin inhibition with MitMAB blocks EGFP-claudin-2 (cyan) endocytosis but does not prevent strand collapse after mCherry-claudin-4 (magenta) expression in U2OS cells. In the absence of claudin-4, dynamin inhibition increases the complexity of EGFP-claudin-2 strand networks. Maximum projection images representative of 4 independent experiments. Scale: 5 µm, 1 µm. **b** STED images show EGFP-claudin-2 in the presence of MitMAB or mCherry-claudin-4, either individually or in combination. Representative of at least 4 independent experiments. Scale: 2 µm. **c** mCherry-claudin-4 (magenta) expression increases EGFP-claudin-2 (cyan) vesicle numbers compared to EGFP-claudin-2 in the absence of claudin-4 expression. Dynamin inhibition using 10 µM MitMAB (dark symbols) reduces claudin-2 vesicle numbers in the presence or absence of claudin-4. *n* = 8–9, representative of 5 independent experiments. 1-way ANOVA. ***P < 0.0001. **d** Fluorescence intensity of EGFP-claudin-2 meshworks is significantly increased by dynamin inhibition. *n* = 7, representative of 5 independent experiments. ***P < 0.0001. **e** Dynamin inhibition increases strand area in the absence of claudin-4 expression. mCherry-claudin-4 induces collapse and reduces the area covered by EGFP-claudin-2 strands, even in the presence of dynamin inhibitor. *n* = 6–8, representative of 5 independent experiments. 1-way ANOVA. **P = 0.0023 (without MitMAB); **P = 0.0032 (with MitMAB); ***P < 0.0001. **f** mCherry-claudin-4 induces collapse of EGFP-claudin-2 networks and reduces numbers of strand intersections. Conversely, dynamin inhibition in the absence of claudin-4 increases EGFP-claudin-2 polymer network complexity. *n* = 8–9, representative of 5 independent experiments. 1-way ANOVA. **P = 0.0012; ***P < 0.0001. **g** Illustration summarizing experimental results. Claudin-4 disrupts claudin-2 strands despite dynamin inhibition. Endocytic blockade in the absence of claudin-4 increases claudin-2 strand numbers, area occupied, and complexity of claudin-2 strand networks. 1-way ANOVA. **P < 0.01; ***P < 0.001. Data are presented as mean ± SD and included in the Source Data file.

Within hours of induction of claudin-4 expression, EGFP-claudin-2 accumulated within intracellular vesicles. This endocytosis was sensitive to myristyltrimethylammonium bromide (MiTMAB) or myristoylated dynamin inhibitory peptide (DIP), two structurally unrelated dynamin inhibitors (Fig. 7a, Supplementary Fig. 3). Endocytosis was also blocked by chlorpromazine (Supplementary Fig. 3), an inhibitor of clathrin-mediated endocytosis, but not by MβCD (Supplementary Fig. 3) or amiloride (Supplementary Fig. 3), which inhibit caveolar endocytosis and macropinocytosis, respectively.

Although it blocked endocytosis, dynamin inhibition did not prevent mCherry-claudin-4 from disrupting claudin-2 strands. Strand networks collapsed into thick structures that also included claudin-4 (Fig. 7a). These changes were best visualized by STED microscopy (Fig. 7b) and could be assessed morphometrically as increased strand fluorescent intensity, decreased strand area, and reduced numbers of nodes where three or more strands intersect (Fig. 7c–f, Supplementary Fig. 3). Thus, claudin-2 endocytosis follows must occur after claudin-4-induced strand disruption. Conversely, dynamin inhibition in the absence of claudin-4 reduced numbers of claudin-2-containing vesicles and greatly increased complexity of claudin-2 strand networks (Fig. 7g), suggesting that endocytosis may be a constitutive mechanism that regulates plasma membrane claudin-2 content and strand architecture.

### Claudin-4 directs claudin-2 removal from epithelial tight junctions

**Fig. 8:**
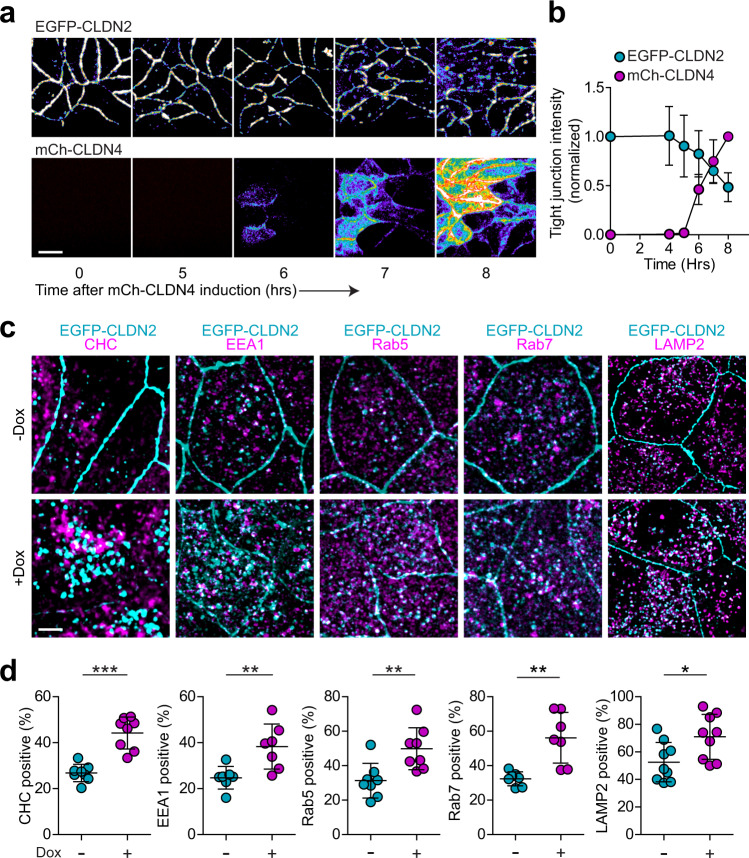
Claudin-4 expression results in clathrin-mediated endocytosis of claudin-2. **a** Live imaging demonstrates progressive EGFP-claudin-2 removal from tight junctions and accumulation in intracellular vesicles after induction of mCherry-claudin-4 expression (both claudins shown as pseudocolor to facilitate visualization of the wide range of intensities). Note the relative preservation of junction-associated EGFP-claudin-2 in regions with lower levels of mCherry-claudin-4 expression, e.g., lower right corner of the last image pair. Representative of at least 4 independent experiments. Scale: 10 µm. **b** Loss of tight junction-associated EGFP-claudin-2 (cyan symbols) correlates temporally with increased mCherry-claudin-4 (magenta symbols) expression. *n* = 4, representative of 3 independent experiments. **c** mCherry-claudin-4 expression (not shown) leads to EGFP-claudin-2 (cyan) endocytosis and colocalization with endocytic markers (magenta) clathrin heavy chain (CHC), EEA1, Rab5, Rab7, and LAMP2. Maximum projection images representative of 3 independent experiments. Scale: 5 µm. **d** The fractions of EGFP-claudin-2 vesicles that contain clathrin heavy chain (CHC, *n* = 8, *P* < 0.0001), early endosome markers EEA1 (*n* = 7, *P* = .007) and Rab5 (*n* = 8, *P* = 0.005), late endosome marker Rab7 (*n* = 7, *P* = 0.0014), or lysosomal marker LAMP2 (*n* = 9, *P* = 0.021) without claudin-4 (−Dox, cyan symbols) is increased by mCherry-claudin-4 expression (+Dox, magenta symbols). Each data point represents 4–5 cells, representative of 3 independent experiments. Two-tail unpaired *t*-test. *P < 0.05, **P < 0.01, **P < 0.0001. Data are presented as mean ± SD and included in the Source Data file.

Within MDCK epithelial monolayers, EGFP-claudin-2 was endocytically removed from tight junctions within hours of mCherry-claudin-4 expression (Fig. 8a, b). This was specific for EGFP-claudin-2, as distributions of other apical junctional complex proteins were unchanged (Supplementary Fig. 5a–d). Once internalized, EGFP-claudin-2 was sequestered within intracellular compartments but was not immediately degraded (Supplementary Fig. 5e, f).

In addition to a 2.1 ± 0.6-fold increase in total numbers of EGFP-claudin-2-positive vesicles (Supplementary Fig. 5e), mCherry-claudin-4 expression increased the fraction of EGFP-claudin-2-containing vesicles that were also positively-stained for clathrin heavy chain, LAMP-2, EEA1, Rab5, and Rab7 (Fig. 8c, d). In contrast, caveolin-1 did not significantly colocalize with vesicular EGFP-claudin-2 (Supplementary Fig. 5g). These data suggest that claudin-4 leads to clathrin-mediated endocytosis of claudin-2 with subsequent trafficking through the endosomal system into lysosomes.

### Endocytic blockade prevents endocytosis but not channel inhibition

**Fig. 9:**
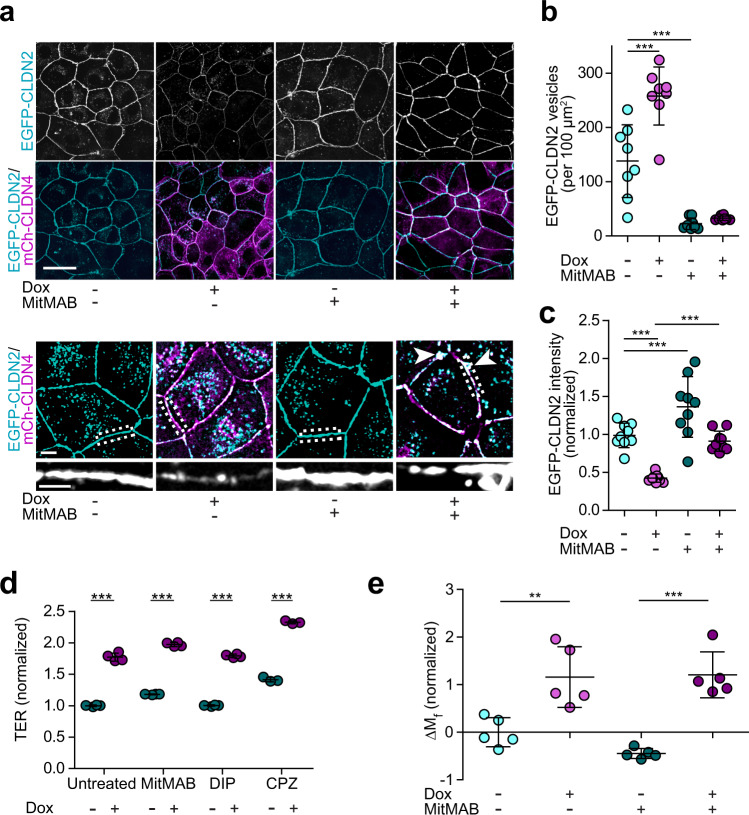
Endocytic inhibition prevents claudin-2 internalization but not channel mobilization or inactivation. **a** The dynamin inhibitor MiTMAB (20 μM) prevents EGFP-claudin-2 (cyan) removal from tight junctions following induction (+Dox) of mCherry-claudin-4 (magenta) expression. Maximum projection images representative of 5 independent experiments. Scale: 20 µm, 5 µm, 2 µm. **b** Dynamin inhibition (dark symbols) reduces numbers of EGFP-claudin-2 vesicles (−Dox, cyan) in the absence of claudin-4 and blocks EGFP-claudin-2 endocytosis triggered by mCherry-claudin-4 expression (+Dox, magenta symbols). *n* = 8. Each data point represents 4–5 cells, representative of 3 independent experiments. 1-way ANOVA. ***P < 0.0001. **c** MiTMAB (dark symbols) increases EGFP-claudin-2 expression at the tight junction in the absence of claudin-4 (−Dox, cyan symbols) and prevents EGFP-claudin-2 endocytosis after mCherry-claudin-4 expression (+Dox, magenta symbols). *n* = 9. Each data point represents 4–5 cells, representative of 5 independent experiments. 1-way ANOVA. ***P < 0.0001. Scale: 5 µm. **d** Endocytic inhibitors (dark symbols) MiTMAB, chlorpromazine (CPZ, 30 μM), or dynamin inhibitory peptide (DIP, 25 μM) do not affect TER in the absence of claudin-4 (cyan symbols) but fail to prevent TER increases induced by mCherry-claudin-4 expression (+Dox, magenta symbols). *n* = 3, representative of 3 independent experiments. Two-tail unpaired *t*-test. ***P < 0.0001. **e** MitMAB (dark symbols) does not affect EGFP-claudin-2 anchoring in the absence of claudin-4 (+Dox, magenta symbols) but is insufficient to prevent EGFP-claudin-2 mobilization after mCherry-claudin-4 expression (+Dox, magenta symbols). *n* = 4, representative of 3 independent experiments. 1-way ANOVA. **P = 0.0063, ***P < 0.001. Data are presented as mean ± SD and included in the Source Data file.

Consistent with clathrin-mediated endocytosis, MiTMAB, DIP, or chlorpromazine each blocked mCherry-claudin-4-induced EGFP-claudin-2 internalization in epithelial cells (Fig. 9a, b), Supplementary Fig. 6a, b). In contrast, neither caveolar endocytosis nor macropinocytosis inhibitors affected mCherry-claudin-4-induced depletion of tight junction-associated EGFP-claudin-2 (Supplementary Fig. 6c, d). Dynamin inhibition in the absence of claudin-4 reduced numbers of EGFP-claudin-2-containing vesicles and increased accumulation at tight junctions, suggesting that, in epithelial cells, surface claudin-2 levels may be regulated by constitutive endocytosis (Fig. 9c).

Despite blocking endocytic removal, dynamin inhibition did not prevent mCherry-claudin-4 from disrupting the normally uniform tight junction distribution of EGFP-claudin-2 (Fig. 9a, lower images). In contrast to the global loss of tight junction-associated claudin-2 typically induced by mCherry-claudin-4 expression (Fig. 8a), the combination of mCherry-claudin-4 expression and dynamin inhibition led to accumulation of EGFP-claudin-2 aggregates at tight junctions (Fig. 9a, lower images).

Although they blocked EGFP-claudin-2 removal from the plasma membrane, neither dynamin nor clathrin-mediated endocytosis inhibitors prevented mCherry-claudin-4-induced TER increases (Fig. 9d). Moreover, dynamin inhibition did not prevent mCherry-claudin-4-induced increases in the EGFP-claudin-2 mobile fraction (Fig. 9e). Together, these data indicate that claudin-4-mediated claudin-2 mobilization and channel inhibition precedes and is independent of endocytosis. The data further suggest that claudin-4-induced increases in claudin-2 mobile fraction reflect disruption of claudin-2 strands similar to that observed in nonepithelial cells.

## Discussion

Tight junctions are essential components of the epithelial barrier. However, tight junctions must also be selectively permeable to ions, water, and macromolecules. Ions and water cross tight junctions via channels created by a subset of claudin proteins while other claudins are thought to form the paracellular barrier^30,32–34^. Here, we studied a prototypic barrier-forming claudin, claudin-4, and found that it is neither required to form nor sufficient to substantively increase the paracellular barrier of MDCK I monolayers. When expressed with pore-forming claudins, however, claudin-4 inhibits channel activity and markedly augments the paracellular barrier. The complete process, which we have termed interclaudin interference, involves simplification and collapse of claudin meshworks, strand depolymerization, and, ultimately, channel disruption. The spectrum of claudin functions must therefore be expanded to include structural and functional regulation via the process of interclaudin interference.

Initially, we found that claudin-4 KO had only minimal effects on barrier function of high resistance MDCK I monolayers. Our result is similar to a previous study that knocked out claudin-4 in low resistance MDCK II cells and observed no change in TER^35^. However, unlike other work, we did not detect any change in Cl^−^ conductance after claudin-4 KO or overexpression, perhaps because claudin-8 is not expressed in MDCK I cells^36,37^. Claudin-4 overexpression also failed to affect TER as well as charge- and size-selectivity, thereby contrasting sharply with previous work^7^. We resolved these discordant results by discovering that claudin-4 only enhances barrier function when cation channel-forming claudins, such as claudins 2 and 15, are expressed. Together with the extremely small changes in Na^+^ conductance induced by claudin-4 KO or overexpression, these data suggest that there may be limited expression of a pore-forming claudin in MDCK I.

Claudin-4 specifically reversed increases in small cation flux induced by expression of claudins 2 or 15. However, channel size was unaffected, suggesting that claudin-4 inhibits a subset of cation channels. As a result of interclaudin interference, claudin-4 expression triggers endocytic removal of claudins 2 and 15 from the tight junction. This contrasts with a prior study work showing that IFN-γ and TNFα increase mobile fractions of both claudin-2 and claudin-4 while reducing the rate of claudin-2 recovery^22^. Moreover, unlike a previous report that claudin-8 can trigger transcriptional repression of claudin-2^38^, we did not detect mCherry-claudin-4-induced changes in EGFP-claudin-2 expression, likely because we used the EF1α promoter to express EGFP-claudin-2. Instead, we found that claudin-4 disrupts higher order claudin-2 and claudin-15 structures, including strands and meshworks. Our data further indicate that this mobilizes claudin-2 and interferes with channel activity prior to endocytosis.

To directly observe higher order claudin structures, we took advantage of a reductionist model in which claudin proteins expressed in nonepithelial cells form polymers that resemble tight junction strands. In contrast to claudins 2, 7, 15, and 19, which formed strands and complex networks, claudin-4 did not form polymers. This result was unexpected, as previous work has shown that claudins 1, 2, 3, 5, 11, 14, 15, and 19 all form strands when expressed in nonepithelial cells^11,28–30,39,40^. Two highly-conserved sites, cis-1 and X-I, within the first extracellular loop (ECL1) have been implicated in claudin polymerization^11^. These sites are, however, conserved in claudin-4 relative to strand-forming claudins. Although claudin-4 does not include tyrosine at the −6 position, which has been shown to enhance affinity of some claudins for the ZO-1 PDZ1 domain^41^, this is not required for strand formation, as claudin-1 forms strands despite lacking tyrosine at that site^19,20,29,30^. Moreover, although PDZ binding is required for efficient delivery to tight junctions^34^, it is not necessary for claudin anchoring at epithelial tight junctions^20^ and is also dispensable for strand formation in nonepithelial cells^28,29^. Current models of claudin polymer assembly are, therefore, insufficient to explain the failure of claudin-4 to form strands. It is nevertheless notable that claudin-4 is one of only a few claudins tested that do not form homotypic interactions as assessed by yeast 2 hybrid assay^37,42^.

Some proteins, including members of the tight junction-associated MARVEL protein (TAMP) family, cannot form strands independently but can be incorporated into, and even regulate the organization of claudin strands^30,43–46^. In contrast, claudin-4 was not incorporated into strands formed by claudins 2, 7, 15, or 19, which may explain the reported short half-life of claudin-4 in epithelial cells^19,47^. Claudin-4 did, however have distinct effects on structures formed by claudins 2, 7, 15 and 19. Claudin-4 also increased mobile fractions of claudins 2, 7, 15, and 19 at epithelial tight junctions, consistent with interclaudin interference. It remains to be determined whether claudin-4 is unique in its ability to regulate structure and function of other claudins.

Our data show that, despite maintenance of cell surface pools by inhibiting endocytosis, claudin-4 disrupts higher order claudin-2 structures, reduces claudin-2 anchoring at the tight junction, and inhibits flux across claudin-2 channels. Previous studies have shown that tight junctions undergo continuous molecular remodeling^20,28,47–49^ and that claudin polymers in nonepithelial cells break, fuse, and add newly-synthesized monomers at breakpoints^28,29^. Taken together, these observations suggest that interclaudin interference may be a result of changes in structural dynamics.

In summary, we have discovered a previously unrecognized mechanism, interclaudin interference, by which claudin structures and ion channels can be negatively regulated by another claudin. Claudin-4 disrupts structures formed by claudins 2, 7, 15, and 19, and enhances barrier function by inhibiting claudin-based cation channels. Interclaudin interference may therefore provide a post-translational means of rapid claudin channel regulation and, potentially, fine-tuning of paracellular permeability.

## Methods

### Cell lines and cell culture

Madin–Darby canine kidney (MDCK) I and MDCK II cells were cultured in low glucose (1 g/L) DMEM was supplemented with 10% fetal bovine serum (FBS) and 15 mM HEPES. U2OS osteosarcoma cells were cultured in high glucose (4.5 g/L) DMEM with 10% FBS and 15 mM HEPES. Doxycycline was used at 10 ng/mL to induce mCherry-claudin-4 expression in MDCK cells. In experiments with endocytic inhibitors, doxycycline was used at 100 ng/mL to accelerate claudin-4 expression in MDCK cells and for all experiments using U2OS cells. All cell lines used were routinely tested and shown to be free of mycoplasma contamination.

### Plasmids and CRISPR KO reagents

EGFP-claudin-2, 7, 15 and 19 were expressed using the EF1α promoter. A PiggyBac vector (System Biosciences) into which a TET-ON3G gene expression system (Clontech) had been incorporated was used for inducible mCherry-claudin-4 expression. Fluorescence-activated cell sorting (FACS Aria, BD Biosciences) was used to sort the top 5–20% GFP-positive cells where polyclonal cell populations were used.

Guide RNA targeting canine *Cldn4* gene exon 2 (5’- GCTGGCCGGCCTGCTGGTCA -3’) was cloned into pSpCas9(BB)-2A-Puro vector and transfected into MDCK I cells. After 5 days of puromycin (10 µg/mL) selection, clones were isolated by limiting dilution in 96-well plates and characterized by immunostaining, western blot, and genomic DNA sequencing.

### Transepithelial electrical resistance (TER) and bionic potential

MDCK cells were plated on 0.33 cm^2^ polycarbonate semipermeable supports (Corning 3413). TER was routinely analyzed 4 days after plating using an epithelial voltohmmeter (EVOM2, World Precision Instruments).

For time course analyses, MDCK cells were plated onto supports which were placed directly into 8-well holders and stations (Advanced Biophysics) within a 37 °C, 5% CO_2_ incubator. The stations were attached to an ECIS ZƟ instrument and cell impedance, Z, was measured using small alternating current at a frequency of 400 Hz. Measurements were recorded continuously and paused briefly in order to replenish the media or add doxycycline.

A series of cations with a range of hydrodynamic diameters (Supplementary Table 1) was used for bi-ionic substitution measurements^50,51^. For dilution potential measurements, buffer with 138 mM NaCl in the basal chamber, was replaced with 69 mM NaCl media iso-osmotically balanced using mannitol. For bi-ionic substitution potential measurements, buffer in the basolateral chamber was replaced with 138 mM M^+^Cl^−^, where M represents each monovalent organic cation. The osmolality of all solutions was balanced with D-mannitol. The ion permeabilities were using potential difference measurements and the Goldman-Hodgkin-Katz equation^50,51^.

### RNA isolation and quantitative real-time PCR

Total RNA was extracted using a RNeasy Mini Kit with on-column DNase I digestion (Qiagen) and quantified by absorbance (Nanodrop 2000). cDNA synthesis was performed using the iScript cDNA reverse-transcriptase kit (Bio-Rad) followed by real-time PCR using gene-specific primers (Supplementary Table 2), SsoAdvanced Universal RT-PCR supermix (Bio-Rad), and a CFX96 thermocycler. C_t_ numbers were normalized to E-cadherin using the ΔΔCt or ΔC_t_ methods, as indicated in each legend.

### Immunostaining

For confocal imaging, immunostaining was performed on MDCK cells grown on semipermeable supports and U2OS cells grown in 8-well chamber slides were fixed in −20 °C methanol and then crosslinked using 0.1 mM bis-(sulfosuccinimidyl) suberate (BS3, ThermoFisher) for 30 min at room temperature^52^. Samples were incubated with primary antibodies for 18 h at 4 °C, washed, incubated with secondary antibodies for 1 h at room temperature, washed, and mounted using #1.5 coverslips and Prolong Antifade Diamond hard setting mounting medium (Invitrogen).

For STED microscopy, U2OS cells that were plated on #1.5H sterile coverslips (Thor Scientific), fixed in 4% paraformaldehyde in PBS for 10 min, and permeabilized with 0.5% Triton-X-100 before antibody incubations. EGFP-claudin-2, 7, 15, and 19 were detected using anti-GFP antibodies.

### Antibodies

Details of all antibodies used are provided in Supplementary Table 3.

### Microscopy

A DM4000 microscope (Leica) with a heated stage, 63X U-V-I NA0.9 dipping objective (Leica), dual emission filter cube (Chroma, 59222), p300 light source (CoolLED) with ET470/40x and ET572/35x excitation filters (Chroma), Rolera EMC2 CCD camera (QImaging), MicroPoint 435 nm dye-tunable guided laser (Photonic Instruments), all controlled by Metamorph 7.9 was used for FRAP. A ~10 µm region of the tight junction was photobleached and then imaged at regular intervals for 600 s, as described^19^. After alignment to correct for cell movement, background was subtracted and mean fluorescence within bleached areas was adjusted to compensate for observation-induced photobleaching using signal at junctions distant from the laser target. Mobile fraction (M_f_) was calculated using the average fluorescence recovery over the final 5 time-points.

Live and fixed cells were imaged on a DMI6000 microscope (Leica) and equipped with a CSU-X1 spinning disk (Yokogawa), motorized xy stage (Ludl), 405, 488, 594 and 660 lasers, multi-band dichroic, individual emission filters (Semrock), 63X/NA1.3 glycerol immersion and 100X/NA 1.4 oil immersion objectives, and Zyla 4.2 cMOS camera (Andor) [within a temperature controlled chamber], controlled by Metamorph 7.9 (Molecular Devices). Postacquisition deconvolution used Autoquant X3 (Media Cybernetics) and analysis made use of Metamorph and ImageJ/FIJI.

STED microscopy used either an Olympus BX83 widefield microscope equipped with a 100X/NA1.4 oil immersion objective (Olympus) and a Facility Line STED system (Abberior Instruments) with a 775 nm depletion laser or a Zeiss Axiovision widefield microscope equipped with a 100X/NA1.4 oil immersion objective (Zeiss) and a STEDYCON system (Abberior Instruments). Both systems were controlled used Imspector software (Abberior).

For live imaging, media was replaced with Fluorobrite DMEM medium (Invitrogen, A1896701) supplemented with 10% FBS and equilibrated at 37 °C, 5% CO_2_ for 30 min to limit photobleaching. Fixed preparations were mounted in ProLong Diamond (Invitrogen).

### Strand/meshwork analysis

Maximum intensity projections of nonepithelial cell claudin strand confocal stacks were analyzed using ImageJ/FIJI (Figs. 5e, 6a–c, and 7e). Vesicle-containing regions were excluded from the analysis, and a threshold was set to cover the strand areas (exemplified in Supplementary Fig. 4a). Area and mean intensities were measured based on a minimum intensity threshold cutoff. The threshold was constant across all conditions within each experiment. Quantification of intersections/nodes from super-resolution STED images was done manually by a blinded observer. Nodes were defined as intersections of at least 3 strands (Supplementary Fig. 4b).

### Western blots

Cells were lysed in RIPA buffer containing 150 mM NaCl, 1% Nonidet P-40, 0.5% sodium deoxycholate, 0.1% SDS in 50 mM Tris, pH 7.4, with Halt protease inhibitor cocktail (ThermoFisher). After denaturation at 98 °C for 5 min and separation on SDS-PAGE gels (Bio-Rad), proteins were transferred to low fluorescence nitrocellulose membranes (Li-Cor), incubated with primary antibodies for 18 h at 4 °C, washed, incubated with infrared dye-conjugated secondary antibodies for 1 h at room temperature, and washed before imaging using an Odyssey Fc imager (Li-Cor). Signals were quantified using ImageStudio 5.0 (Li-Cor). Uncropped blots (Figs. 1b, 2a and Supplementary Fig. 5e) are provided in the source data file.

### Statistics and reproducibility

All data, including imaging studies, are representative of at least three independent experiments. In graphs, data are presented as mean ± SD. Statistical significance was determined by two-tailed Student’s *t* test (unpaired) or ANOVA, as indicated, using GraphPad Prism 9. Absolute *P* values are indicated each figure legend.

### Reporting summary

Further information on research design is available in the Nature Research Reporting Summary linked to this article.

## Supplementary information

Supplementary Information

Reporting Summary

## Data Availability

Source data, methods, and analysis data are provided in the paper and supplemental files. Further information regarding this paper is available from the lead contact upon request. Source data are provided with this paper.

## Source data

Source Data
