# Supplementary material for: Tight junction channel regulation by interclaudin interference
Source: Nat Commun. 2022 Jun 30;13:3780. doi: 10.1038/s41467-022-31587-8 (PMC9246906; doi:10.1038/s41467-022-31587-8)
Supplement: Supplementary file 1 — Supplementary Information [file 41467_2022_31587_MOESM1_ESM.pdf]

SUPPLEMENTARY INFORMATION

Tight junction channel regulation by interclaudin interference

Nitesh Shashikanth[1], Marion M. France[1], Ruyue Xiao[1], Xenia Haest[1],
Heather E. Rizzo[1], Jose Yeste[1,2], Johannes Reiner[3], and Jerrold R. Turner[1,*]

[1] Laboratory of Mucosal Barrier Pathobiology, Department of Pathology, Brigham and Women's Hospital and Harvard Medical School, Boston, Massachusetts, USA

[2] Instituto de Microelectrónica de Barcelona, IMB-CNM (CSIC), Bellaterra, Spain

[3] Division of Gastroenterology and Endocrinology, Department of Medicine II, Rostock University Medical Center, Ernst-Heydemann-Str. 6, Rostock, Germany

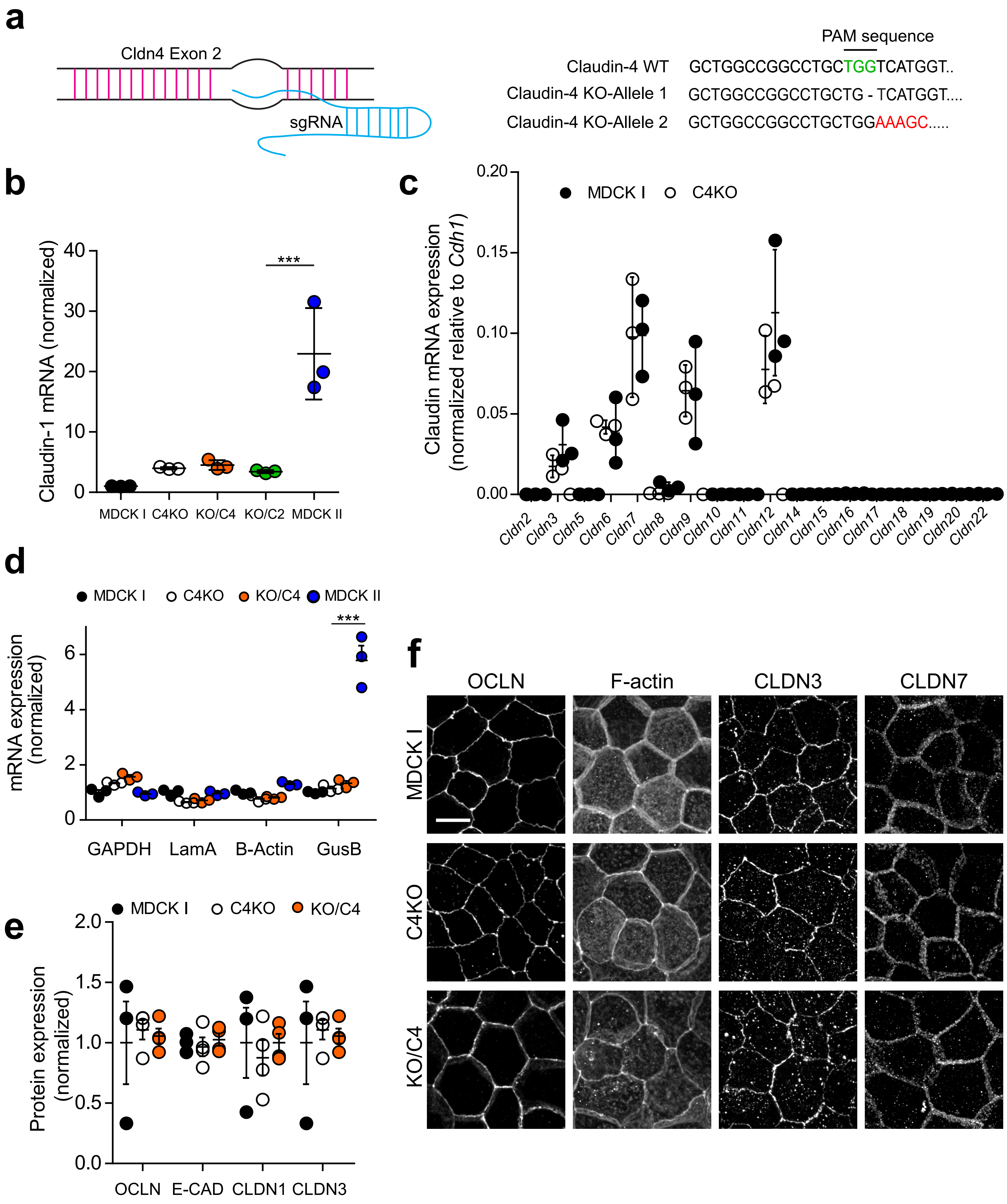
a
Cldn4 Exon 2
sgRNA
PAM sequence
Claudin-4 WT GCTGGCCGGCCTGCTGGTCATGGT..
Claudin-4 KO-Allele 1 GCTGGCCGGCCTGCTG - TCATGGT....
Claudin-4 KO-Allele 2 GCTGGCCGGCCTGCTGGAAAGC.....
b
Claudin-1 mRNA (normalized)
***
MDCK I C4KO KO/C4 KO/C2 MDCK II
c
Claudin mRNA expression (normalized relative to Cdh1)
MDCK I C4KO
Cldn2 Cldn3 Cldn5 Cldn6 Cldn7 Cldn8 Cldn9 Cldn10 Cldn11 Cldn12 Cldn14 Cldn15 Cldn16 Cldn17 Cldn18 Cldn19 Cldn20 Cldn22
d
MDCK I C4KO KO/C4 MDCK II
mRNA expression (normalized)
***
GAPDH LamA B-Actin GusB
e
MDCK I C4KO KO/C4
Protein expression (normalized)
OCLN E-CAD CLDN1 CLDN3
f
OCLN F-actin CLDN3 CLDN7
MDCK I
C4KO
KO/C4

Supplementary Fig. 1 Characterization of CRISPR-mediated claudin-4 KO MDCK I cells. **a** Guide RNA was designed to specifically target genomic DNA encoding claudin-4. Genomic sequencing showing frameshift and premature termination insertions. **b** Claudin-1 mRNA expression is non-significantly increased, relative to WT MDCK I (black symbols) in claudin-4 KO (white symbols). Results are similar in three independent clones. Claudin-1 transcription is not affected by mCherry-claudin-4 (orange symbols) or EGFP-claudin-2 (green symbols) expression. Claudin-1 expression in MDCK I and all MDCK I-derived clones is far less than that of WT MDCK II (blue symbols). Data normalized to MDCK I. n = 3, representative of 3 independent experiments. 1-way ANOVA. ***, P<0.0001. **c** qRT-PCR analyses of WT and C4KO show similar expression of all claudins except claudin-1. Data normalized to E-cadherin (ΔC_t). n = 3, representative of 3 independent experiments. Two-tailed unpaired t-test. **d** mRNA expression of housekeeping genes relative to E-cadherin in MDCK I (black symbols), claudin-4 KO (white symbols), claudin-4 KO overexpressing claudin-4 (orange symbols), or MDCK II (blue symbols). Data normalized to MDCK I. n = 3, representative of 3 independent experiments. 1-way ANOVA. ***, P<0.0001. **e** Densitometry of SDS-PAGE immunoblots show that claudin-1 (CLDN1), claudin-3 (CLDN3), occludin (OCLN) and E-cadherin (E-CAD) protein expression are unaffected by claudin-4 KO or overexpression. n = 3-4, representative of 3 independent experiments. Data normalized to MDCK I. 1-way ANOVA. A representative immunoblot is shown in figure 1B. **f** Maximum projection images show that distributions of OCLN, F-actin, CLDN3, and CLDN7 are not affected by claudin-4 KO or overexpression. Scale: 10 µm. Data are presented as mean ± SD and included in the Source Data file.

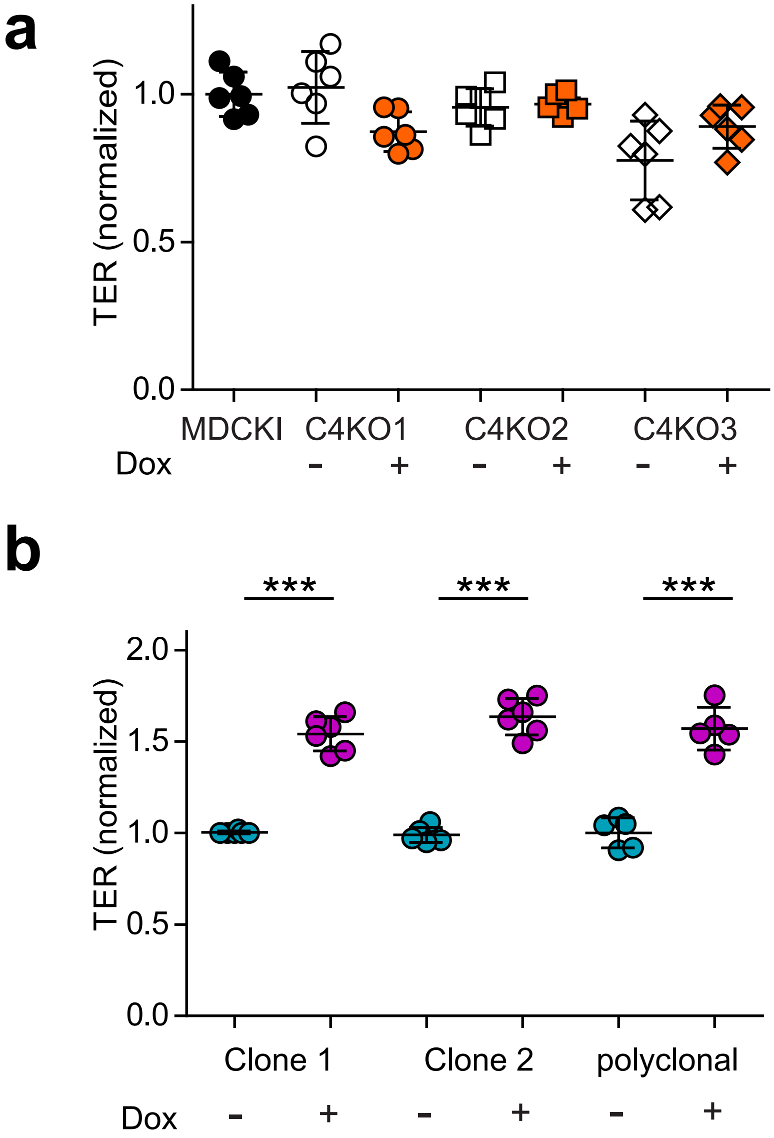
a
TER (normalized)
0.0
0.5
1.0
MDCKI
C4KO1
C4KO2
C4KO3
Dox
-
+
-
+
-
+
b
***
***
***
TER (normalized)
0.0
0.5
1.0
1.5
2.0
Clone 1
Clone 2
polyclonal
Dox
-
+
-
+
-
+

Supplementary Fig. 2 a Peak TER of MDCK I (black symbols) and three independent MDCK I claudin-4 KO clones without (-Dox, white symbols) or with (+Dox, orange symbols) inducible mCherry-claudin-4 overexpression. Neither claudin-4 KO nor mCherry-claudin-4 overexpression significantly affected TER. n = 6, representative of 4 independent experiments. 2-tail unpaired t-test. **b** TER of two monoclonal MDCKI lines with constitutive EGFP-claudin-2 expression without (-Dox, cyan symbols) or with (+Dox, magenta symbols) induction of mCherry-claudin-4 expression. These clones were created by transfecting separate claudin-4 KO clones with vectors encoding EGFP-claudin-2 and mCherry-claudin-4. The third dataset shows a clonal claudin-4 KO line with inducible mCherry-claudin-4 expression that was transfected to express EGFP-claudin-2 constitutively. Cell sorting was used to obtain a polyclonal population of EGFP-claudin-2-expressing cells. n = 5-6, representative of 3 independent experiments. Two-tail unpaired t-test. ***, $P<0.0001$. Data are presented as mean ± SD and included in the Source Data file.

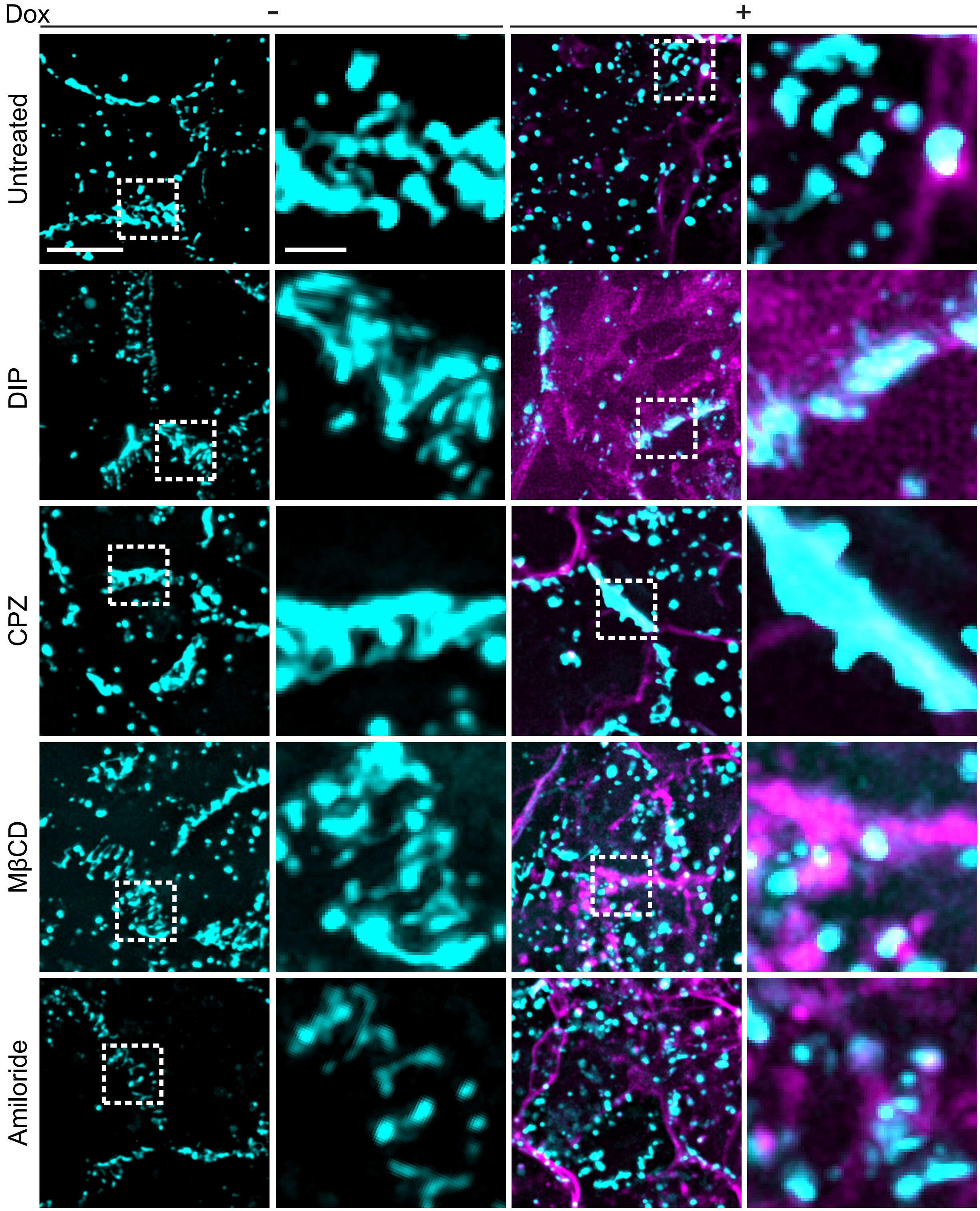

Dox
-
+
Untreated
DIP
CPZ
MβCD
Amiloride

Supplementary Fig. 3 Inhibition of clathrin-mediated endocytosis, but not caveolar endocytosis or macropinocytosis, prevents mCherry-claudin-4-induced EGFP-claudin-2 internalization. U2OS cells with constitutive EGFP-claudin-2 (cyan) expression without (-Dox) or with (+Dox) induction of mCherry-claudin-4 (magenta). Insets show presence and absence of EGFP-claudin-2 strands before and after mCherry-claudin-4 expression, respectively. Myristoylated dynamin inhibitory peptide (DIP) or chlorpromazine (CPZ), an inhibitor of clathrin-mediated endocytosis, both prevent EGFP-claudin-2 internalization but fail to block EGFP-claudin-2 strand collapse (arrowheads) after mCherry-claudin-4 expression. Neither the cholesterol-binding caveolar endocytosis inhibitor methyl-β-cyclodextrin (MβCD) nor amiloride, which blocks macropinocytosis, prevents claudin-4-induced endocytosis of EGFP-claudin-2. Scale: 10 µm, zoomed insets: 2 µm. Images are representative of 3 independent experiments.

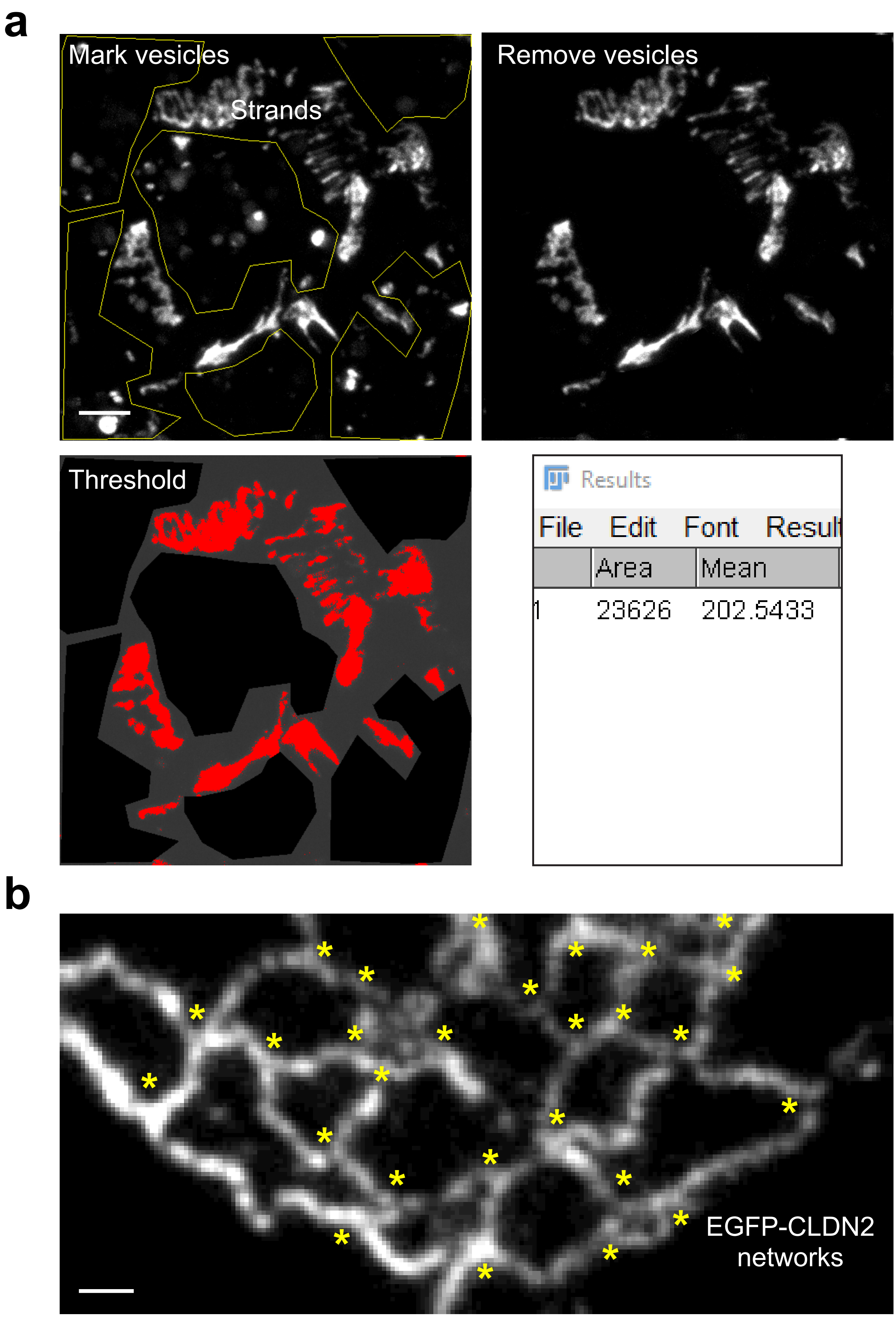
a
Mark vesicles
Strands
Remove vesicles
Threshold
Results
File Edit Font Result
Area Mean
1 23626 202.5433
b
EGFP-CLDN2
networks

Supplementary Fig. 4 Morphometric approach. **a** Maximum projection confocal image of U2OS cells expressing EGFP-claudin-2. Note the presence of strands and vesicles (intracellular, circular dull or bright spots). The vesicle containing regions were excluded from the analysis, and a threshold was set to cover the strand areas. Area and mean intensities of pixels were measured based on the threshold cutoff using ImageJ/FIJI. **b** A patch of STED-resolved EGFP-claudin-2 strands with the nodes marked as yellow asterisks. In this example, there are 26 nodes where at least 3 strands intersect. Scale A: 5 µm, B: 500 nm.

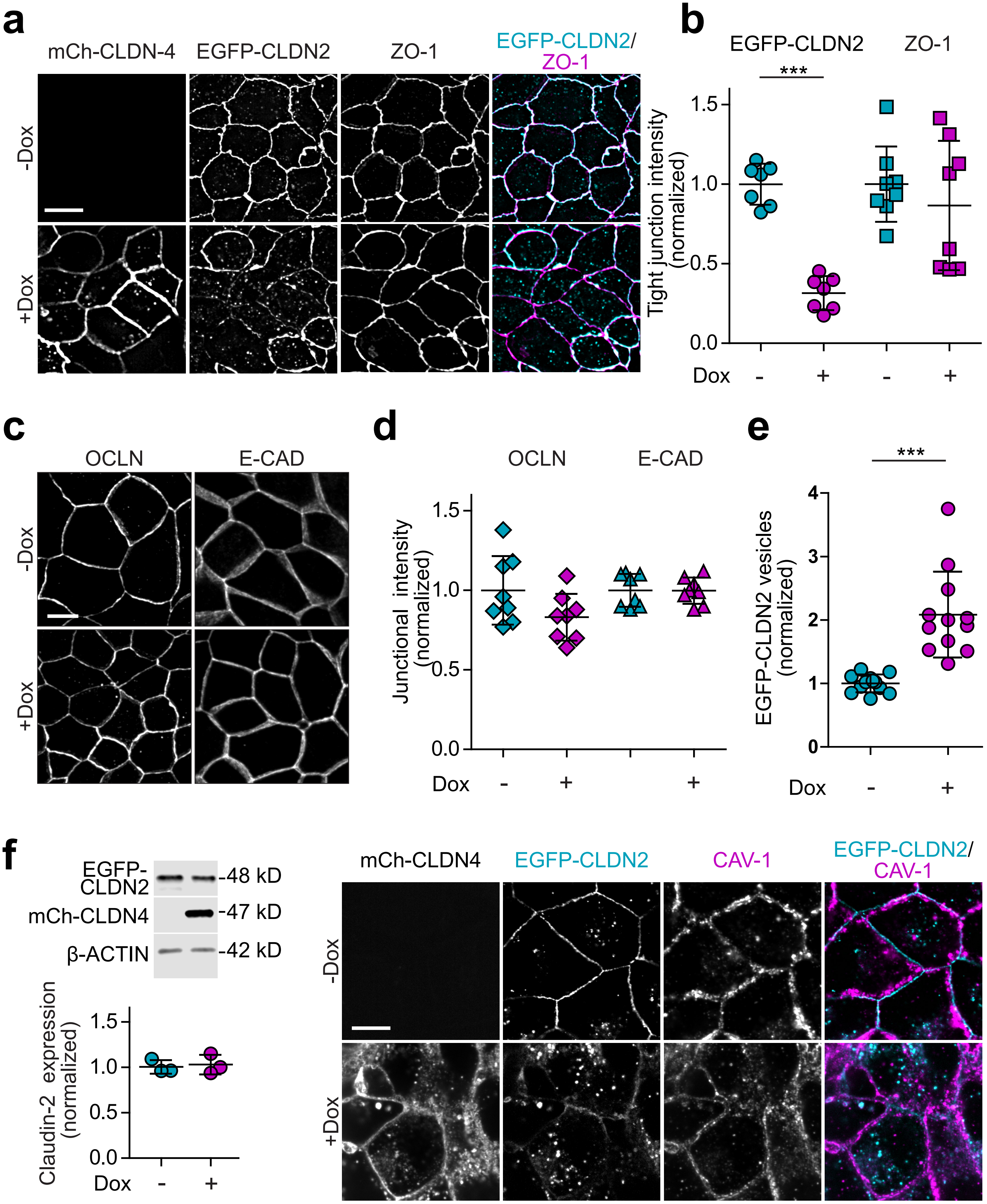
a
mCh-CLDN-4
EGFP-CLDN2
ZO-1
EGFP-CLDN2/
ZO-1
-Dox
+Dox
b
EGFP-CLDN2
ZO-1
***
Tight junction intensity
(normalized)
1.5
1.0
0.5
0.0
Dox
-
+
-
+
c
OCLN
E-CAD
-Dox
+Dox
d
OCLN
E-CAD
Junctional intensity
(normalized)
1.5
1.0
0.5
0.0
Dox
-
+
+
e
***
EGFP-CLDN2 vesicles
(normalized)
4
3
2
1
0
Dox
-
+
f
EGFP-
CLDN2
-48 kD
mCh-CLDN4
-47 kD
β-ACTIN
-42 kD
Claudin-2 expression
(normalized)
1.5
1.0
0.5
0.0
Dox
-
+
mCh-CLDN4
EGFP-CLDN2
CAV-1
EGFP-CLDN2/
CAV-1
-Dox
+Dox

Supplementary Fig. 5 Claudin-4 directs claudin-2 removal from tight junctions. **a** Maximum projection images of EGFP-claudin-2, mCherry-claudin-4, and ZO-1. Only EGFP-claudin-2 (cyan) and ZO-1 (magenta) are shown in the merged color image. Images representative of 5 independent experiments. **b** Despite loss of EGFP-claudin-2 (circles), tight junction-associated ZO-1 (squares) levels were similar before (cyan symbols) and after (magenta symbols) mCherry-claudin-4 expression. n = 8 regions consisting of 7-8 cells, representative of 5 independent experiments. 2-tail unpaired t-test. ***, $P<0.0001$. **c, d** Distributions and fluorescent intensities of occludin (OCLN, diamonds) or E-cadherin (E-CAD, triangles) in EGFP-claudin-2-expressing monolayers are similar in the absence (cyan) or presence (magenta) of mCherry-claudin-4 expression. n = 8 regions comprising 7-8 cells, representative of 3 independent experiments. **e, f** mCherry-claudin-4 induces endocytosis, but not degradation of EGFP-claudin-2. EGFP-CLDN2 vesicles counts show n = 8 regions consisting of 4-5 cells, representative of 5 independent experiments. Two-tail unpaired t-test. ***, $P <0.0001$. Western blot shows n = 3, representative of 5 independent experiments. Two-tail unpaired t-test. Maximum projection images show that the overall distribution of caveolin-1 (CAV-1) is not affected by mCherry-claudin-4 expression. Caveolin-1 staining of EGFP-claudin-2 vesicles is minimal before and after claudin-4 expression. EGFP-claudin-2 (cyan) and caveolin-1 (magenta) are shown in the merged color image. Scale: 10 µm. Data are presented as mean ± SD and included in the Source Data file.

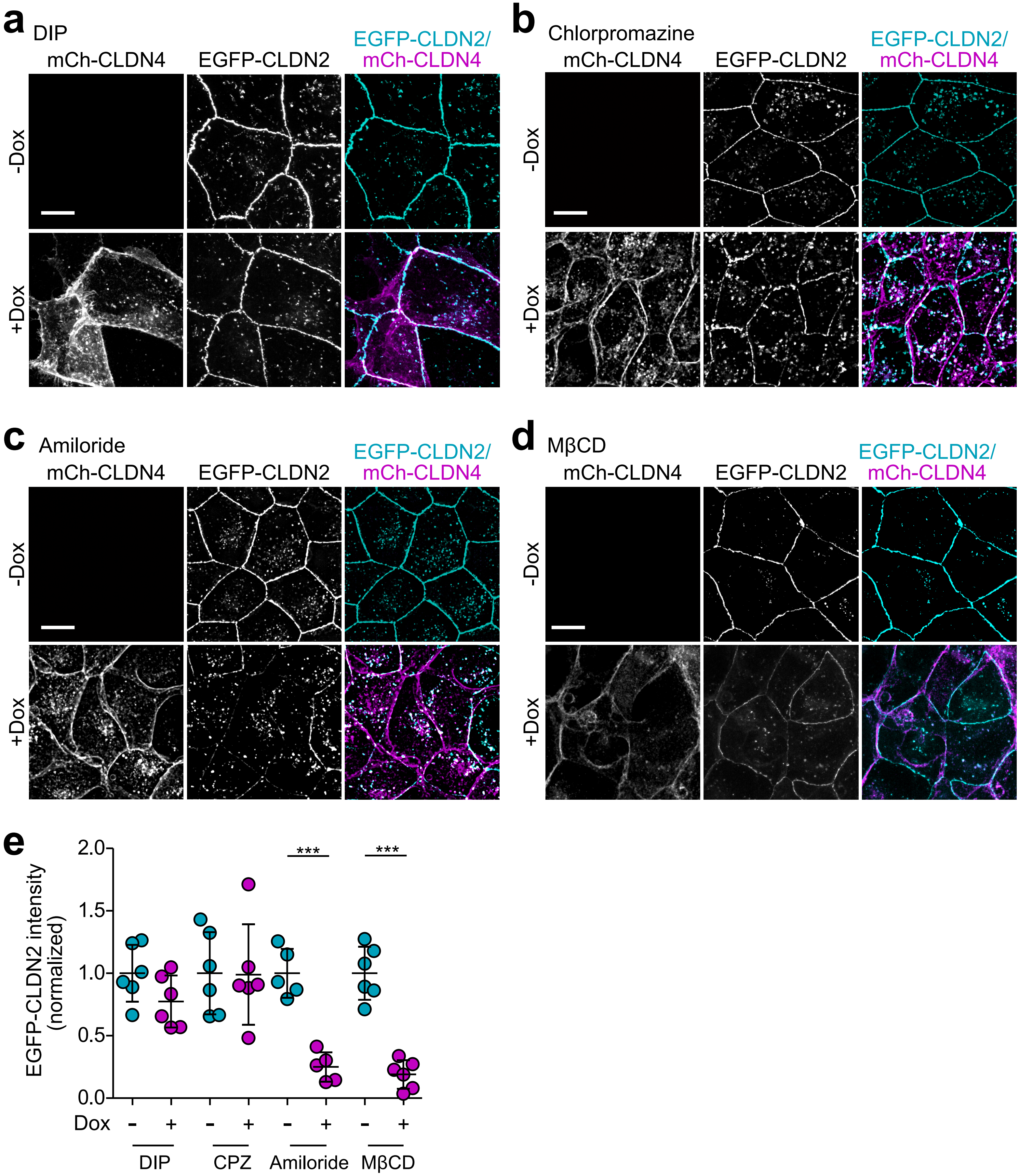

a
DIP
mCh-CLDN4
EGFP-CLDN2
EGFP-CLDN2/
mCh-CLDN4
-Dox
+Dox
b
Chlorpromazine
mCh-CLDN4
EGFP-CLDN2
EGFP-CLDN2/
mCh-CLDN4
-Dox
+Dox
c
Amiloride
mCh-CLDN4
EGFP-CLDN2
EGFP-CLDN2/
mCh-CLDN4
-Dox
+Dox
d
MβCD
mCh-CLDN4
EGFP-CLDN2
EGFP-CLDN2/
mCh-CLDN4
-Dox
+Dox
e
EGFP-CLDN2 intensity
(normalized)
2.0
1.5
1.0
0.5
0.0
***
***
Dox
- + - + - + - +
DIP
CPZ
Amiloride
MβCD

Supplementary Fig. 6 Inhibition of clathrin-mediated endocytosis preserves junction-associated claudin-2. **a, b, c, d** Treatment of MDCK cells expressing EGFP-claudin-2 with the dynamin inhibitory peptide DIP (25 μM) or the clathrin-mediated endocytosis inhibitor chlorpromazine (CPZ, 30 μM) prevents claudin-2 from being internalized after induction of mCherry-claudin-4. In contrast, neither the macropinocytosis inhibitor amiloride (5 μM) nor the caveolar endocytosis inhibitor methyl-β-cyclodextrin (MβCD, 2 mM) prevent claudin-4-induced removal of claudin-2 from the tight junctions. Maximum projection images, representative of 3 independent experiments. The merged image shows EGFP-claudin-2 (cyan) and mCherry-claudin-4 (magenta). Scale: 10 μm. **e** Morphometry of EGFP-claudin-2 intensity at tight junctions before (-Dox, cyan symbols) and after (+Dox, magenta symbols) mCherry-claudin-4 expression in the presence of endocytic inhibitors. n = 5-6 points consisting of 4-5 cells, representative of 3 independent experiments. Two-tail unpaired t-test. ***, $P < 0.0001$. Data are presented as mean ± SD and included in the Source Data file.

Supplementary Table 1: Cations used for bi-ionic potentials

**Abbreviation**	**Cation$^+$**	**Dia. (Å)**
Na^+	Sodium	1.90
MA	Methylamine	3.78
EA	Ethylamine	4.58
TMA	Tetramethylamine	5.50
TEA	Tetraethylamine	6.58
NMDG	N-Methyl D-Glucosamine	7.29

Supplementary Table 2: qRT-PCR primers for measuring canine gene transcripts.

Target gene	Forward Primer (5'-3')	Reverse Primer (5'-3')
Cldn1	ATGGAAGACGATGAGGTGC	GCAACTAAAACAGCCAGACC
Cldn2	CCACTCCAGAGCCATACAGC	TCAGAGTGTCTCTGCCAAGC
Cldn3	ACCAAGATCGTCTACTCCGC	CCCCTCACACGTAGTCCTTG
Cldn4	AAGTGAAAGTCTTCCCTGTGG	ATGCCCATCACCTGCAG
Cldn5	TGCAGCTCCTGAAGTGG	GCACAGCCTCGGATCTG
Cldn6	GCGTGGAAGACAAGGACTC	GGACCCCTGAGATGACAAAG
Cldn7	TTGTCACGGACTTCTACAACC	CTTTGCTCTCGCTCCCAG
Cldn8	GCCTAACACCCATCTCGG	TCCAATGAAGGCAGACACTC
Cldn9	CTTCATCGGCAACAGCATC	GAGTCGTACACCTTGCACTG
Cldn10	ACCAGGGTCTGTGGATGAAC	AGCCTCCGACTTTGGTACAC
Cldn11	CAGGTTACATTTTGCTGGTCG	GTGGTAGGGACGGTTGC
Cldn12	ATCTCTACTGTCTATTCAGATGTGC	GGACTGTGGCTGCATGGAC
Cldn13	AGAACTTCTACAACCCGCTG	AGGAGATGAAGCCGAGGTAG
Cldn14	CTCTGGTTTAGCTGTGCCACC	CAGGCCTGGAGGTACCCAGAGA
Cldn16	TTTTCTCTACTGGGTTCTTGGTC	GTGCGAATCCCATCAAAAGC
Cldn17	TCTGGTTCTTGGGTTCTTTGG	AAAAGCTGATACCCTCCACTG
Cldn18	CGAGAGCTCTGGCTTCACCGA	GCACTTCAGGGCAAAGATGGA
Cldn19	GCAAGCTCTATGACTCCCTG	TTCATGCCGACCACACTG
Cldn20	AAGGTGAATGTGGATTCGGG	GAGGGACAGAATGGAGAACTTC
Cldn22	AAATGGAGAACTGGACCGTG	GTTGGACAGGAGCATGAGG
E-cad	CAAGGATCTGTCACGGAAGG	GGCAGCGTTGTAGGTATTCA
Gapdh	AAGGTCATCCCTGAGCTGAA	GATGCCTGCTTCACTACCTT
β-Actin	CGACAGGATGCAGAAGGAAA	TTGATCTTCATCGTGCTGGG
LMNA	CTATCGCTTCCCACCAAAGT	GCCTTCCATACCAAGTCAGTAG
GusB	GAAGCCTGCTGCTTACTACTT	CCGTACTCGCTCTGGATTATTG

Supplementary Table 3: Antibodies used in all studies.

Antibody	Source	Catalog/RRID	Dilution used
Mouse monoclonal anti-β-actin	Abcam	Cat. ab6276, Lot GR181659-14, RRID:AB_2223210	1:5000, 0.5μg/mL
Mouse monoclonal anti-dog- LAMP2	Bio-Rad	Cat. MCA2293GA, Lot 1701, RRID:AB_2134762	1:1000, 1μg/mL
Rabbit monoclonal anti-E-cadherin	Cell Signaling Technologies	Cat# 24E10-3195, Lot 13, RRID:AB_2291471	1:1000, 0.5μg/mL
Mouse monoclonal anti-claudin-1	Invitrogen	Cat. 37-4900, Lot VH307516, RRID: AB_2533323	1:500, 1μg/mL
Mouse monoclonal anti-claudin-2	Invitrogen	Cat. 32-5600, Lot VC297814, RRID:AB_86980	1:500, 1μg/mL
Mouse monoclonal anti-claudin-4	Invitrogen	Cat. 32-9400, Lot TI275403, RRID:AB_86919	1:500, 1μg/mL
Mouse monoclonal anti-GFP	DSHB, Iowa	Clone GFP-G1, RRID:AB_2619561	1:20 (supernatant)
Mouse monoclonal anti-GFP	DSHB, Iowa	Cat# DSHB-GFP-Clone 12E6, RRID:AB_2617418	1:20 (supernatant)
Mouse monoclonal anti-occludin	Invitrogen	Cat. 33-1500, Lot 1578827A, RRID:AB_2533101	1:1000, 0.5μg/mL
Mouse monoclonal anti-ZO1	Invitrogen	Cat. 33-9100, Lot QC215031, RRID:AB_2533147	1:1000, 0.5μg/mL
Mouse monoclonal anti-caveolin-1	Invitrogen	Cat. 03-6000, Lot 40487374, RRID:AB_2532932	1:1000, 0.5μg/mL
Mouse monoclonal anti-clathrin heavy chain	Invitrogen	Cat. MA1-065 X22, RRID: AB_2083179	1:1000, 0.5μg/mL
Rabbit polyclonal anti-claudin-3	Invitrogen	Cat. 34-1700, Lot QO215616	1:500, 0.5μg/mL
Rabbit polyclonal anti-claudin-7	Invitrogen	Cat. 34-9100, Lot Gr317802-4, RRID:AB_2533190	1:500, 0.5μg/mL
Rabbit polyclonal anti-EEA-1	Abcam	Ab2900, Lot GR3288565-1, RRID:AB_2262056	1:300, 1μg/mL
Rabbit polyclonal anti-Rab-5	Cell Signaling Technology	Cat #3547, Lot 7, RRID:AB_2300649	1:200, 0.23μg/mL
Rabbit polyclonal anti-	Cell Signaling	Cat #9367, Lot 3,	1:50, 0.4μg/mL

Rab-7	Technology	RRID:AB_1904103	
Rat monoclonal anti-occludin	J.R. Turner	Clone 6B8A3, RRID: AB_2819194	1:2000, 0.5μg/mL
Rat monoclonal anti-ZO1	D. Goodenough	Clone R40.76, RRID:AB_2783859	1:800, 1μg/mL
Abberior STAR RED goat anti-mouse IgG	Abberior	Cat #STRED-1001-20UG, Lot 10127PK-2	1:1000, 1μg/mL
IRDye 800CW goat anti-rabbit IgG	LI-COR Biosciences	Cat. 925-32211, Lot C70918-02, RRID: AB_2651127	1:10000, 0.1μg/mL
IRDye 680LT goat anti-rabbit IgG	LI-COR Biosciences	Cat. 926-68021, Lot C70901-05, RRID: AB_ 10706309	1:10000, 0.1μg/mL
IRDye 680RD-goat anti-mouse IgG	LI-COR Biosciences	Cat. 926-68070, Lot C70214-04, RRID: AB_2651128	1:10000, 0.1μg/mL
IRDye 800CW-goat anti-mouse IgG	LI-COR Biosciences	Cat. 926-32210, Lot D01110-03, RRID AB_621842	1:10000, 0.1μg/mL
Alexa 647 donkey anti-rat IgG highly cross-adsorbed F(ab')2 fragments	Jackson ImmunoResearch	Cat. 712-606-153; Lot 159129, RRID:AB_2340696	1:1000, 1.5μg/mL
Alexa 647-donkey anti-mouse IgG highly cross-adsorbed F(ab')2 fragments	Jackson ImmunoResearch	Cat. 715-606-151; Lot 156413, RRID:AB_2340866	1:1000, 1.5μg/mL
Alexa 647-donkey anti-rabbit IgG highly cross-adsorbed F(ab')2 fragments	Jackson ImmunoResearch	Cat. 711-606-152; Lot 151523, RRID:AB_2340625	1:1000, 1.5μg/mL
