# Supplementary material for: Tight junction channel regulation by interclaudin interference
Source: Nat Commun. 2022 Jun 30;13:3780. doi: 10.1038/s41467-022-31587-8 (PMC9246906; doi:10.1038/s41467-022-31587-8)
Supplement: Supplementary file 2 — Reporting Summary [file 41467_2022_31587_MOESM2_ESM.pdf]

nature portfolio

Corresponding author(s): Jerrold R. Turner
Last updated by author(s): Apr 5, 2022

# Reporting Summary

Nature Portfolio wishes to improve the reproducibility of the work that we publish. This form provides structure for consistency and transparency in reporting. For further information on Nature Portfolio policies, see our Editorial Policies and the Editorial Policy Checklist.

## Statistics

For all statistical analyses, confirm that the following items are present in the figure legend, table legend, main text, or Methods section.

| n/a | Confirmed | |
|---|---|---|
| ☐ | ☒ | The exact sample size (*n*) for each experimental group/condition, given as a discrete number and unit of measurement |
| ☐ | ☒ | A statement on whether measurements were taken from distinct samples or whether the same sample was measured repeatedly |
| ☐ | ☒ | The statistical test(s) used AND whether they are one- or two-sided<br>*Only common tests should be described solely by name; describe more complex techniques in the Methods section.* |
| ☒ | ☐ | A description of all covariates tested |
| ☐ | ☒ | A description of any assumptions or corrections, such as tests of normality and adjustment for multiple comparisons |
| ☐ | ☒ | A full description of the statistical parameters including central tendency (e.g. means) or other basic estimates (e.g. regression coefficient) AND variation (e.g. standard deviation) or associated estimates of uncertainty (e.g. confidence intervals) |
| ☐ | ☒ | For null hypothesis testing, the test statistic (e.g. *F*, *t*, *r*) with confidence intervals, effect sizes, degrees of freedom and *P* value noted<br>*Give P values as exact values whenever suitable.* |
| ☒ | ☐ | For Bayesian analysis, information on the choice of priors and Markov chain Monte Carlo settings |
| ☒ | ☐ | For hierarchical and complex designs, identification of the appropriate level for tests and full reporting of outcomes |
| ☒ | ☐ | Estimates of effect sizes (e.g. Cohen's *d*, Pearson's *r*), indicating how they were calculated |

*Our web collection on statistics for biologists contains articles on many of the points above.*

## Software and code

Policy information about availability of computer code

| | |
|---|---|
| Data collection | Metamorph (molecular devices) was used to run our widefield deconvolution and confocal microscopes (confocal). Imspector software provided by the manufacture (Abberior GmbH), used to run the super resolution STED microscope. |
| Data analysis | Metamorph and ImageJ/FIJI (NIH) were used for image analysis with only their in-built macros and journals. Autoquant X3 (Media Cybernetics) was used to deconvolve images. All are commercial software. |

For manuscripts utilizing custom algorithms or software that are central to the research but not yet described in published literature, software must be made available to editors and reviewers. We strongly encourage code deposition in a community repository (e.g. GitHub). See the Nature Portfolio guidelines for submitting code & software for further information.

## Data

Policy information about availability of data

All manuscripts must include a data availability statement. This statement should provide the following information, where applicable:

- Accession codes, unique identifiers, or web links for publicly available datasets
- A description of any restrictions on data availability
- For clinical datasets or third party data, please ensure that the statement adheres to our policy

Source data, methods, and analysis are provided in the paper and supplemental files. Further information is available from the lead contact upon request.

# Field-specific reporting

Please select the one below that is the best fit for your research. If you are not sure, read the appropriate sections before making your selection.

☒ Life sciences ☐ Behavioural & social sciences ☐ Ecological, evolutionary & environmental sciences

For a reference copy of the document with all sections, see nature.com/documents/nr-reporting-summary-flat.pdf

# Life sciences study design

All studies must disclose on these points even when the disclosure is negative.

| | |
|---|---|
| Sample size | All sample sizes were at least n=3 for each condition, with representative data shown in the manuscript. All experiments were performed at least three or more times to confirm results. All the sample sizes are indicated in the relevant figure legends in the manuscript. |
| Data exclusions | no data was excluded |
| Replication | Experiments were repeated successfully at least 3 times. |
| Randomization | Not relevant to this study- all reagents and cell lines created are used only after verification of development of the reagent, thus cannot be randomized. |
| Blinding | For manual morphometric analyses observers were blinded to the experimental condition. |

# Reporting for specific materials, systems and methods

We require information from authors about some types of materials, experimental systems and methods used in many studies. Here, indicate whether each material, system or method listed is relevant to your study. If you are not sure if a list item applies to your research, read the appropriate section before selecting a response.

## Materials & experimental systems

| n/a | Involved in the study |
|---|---|
| ☐ | ☒ Antibodies |
| ☐ | ☒ Eukaryotic cell lines |
| ☒ | ☐ Palaeontology and archaeology |
| ☒ | ☐ Animals and other organisms |
| ☒ | ☐ Human research participants |
| ☒ | ☐ Clinical data |
| ☒ | ☐ Dual use research of concern |

## Methods

| n/a | Involved in the study |
|---|---|
| ☒ | ☐ ChIP-seq |
| ☒ | ☐ Flow cytometry |
| ☒ | ☐ MRI-based neuroimaging |

## Antibodies

Antibodies used

All antibodies used in this paper are tabulated in the methods section of the manuscript with all relevant details including dilutions, as well as the validation details below.

Validation

Each antibody has its specific Research Resource Identifiers (RRID) number enlisted in the manuscript. Primary Antibody Source Catalog/Lot Verification/Validation Link to manufacturer:
Mouse monoclonal anti-β-actin. Abcam Cat. ab6276, RRID:AB_2223210. knockout validated by manufacturer. https://www.abcam.com/beta-Actin-antibody-AC-15-ab6276.html?gclsrc=aw.ds|aw.ds&gclid=Cj0KCQjw1ouKBhC5ARIsAHXNMI97SMXv22_CcDJT0bUgGb5xoL8xHxc1LAgS3UffjzJsR9ty0vjZSasaAqdkEALw_wcB
Mouse monoclonal anti-dog- LAMP2 Biorad Cat. MCA2558GA, clone AC17. RRID: AB_1055596. Nabi IR, Le Bivic A, Fambrough D, Rodriguez-Boulan E. An endogenous MDCK lysosomal membrane glycoprotein is targeted basolaterally before delivery to lysosomes. J Cell Biol 1991;115:1573-84. PMID 1757463. PMC2289220. https://www.bio-rad-antibodies.com/monoclonal/dog-canine-cd107b-antibody-ac17-mca2558.html?f=purified
Rabbit monoclonal anti-E-cadherin Cell Signaling technologies Cat# 24E10-3195, RRID:AB_2291471. validated by manufacturer. https://www.cellsignal.com/products/primary-antibodies/e-cadherin-24e10-rabbit-mab/3195
Mouse monoclonal anti-claudin-1, Invitrogen (clone-2H10D10), Cat# 37-4900; RRID: AB_2533323. verified by cell treatment. https://www.thermofisher.com/antibody/product/Claudin-1-Antibody-clone-2H10D10-Monoclonal/37-4900
Mouse monoclonal anti-claudin-2 Invitrogen Cat. 32-5600, RRID:AB_86980. Our data show that this antibody does not label MDCK I cells (which do not express MDCK I) but does label MDCK II cells. https://www.thermofisher.com/antibody/product/Claudin-2-Antibody-clone-12H12-Monoclonal/32-5600
Mouse monoclonal anti-claudin-4, Invitrogen Cat. 32-9400, clone 3E2C1; RRID:AB_86919. Our data show that this antibody does not label claudin-4 KO MDCK I cells but does label MDCK I cells. https://www.thermofisher.com/antibody/product/Claudin-4-Antibody-clone-3E2C1-Monoclonal/32-9400
Mouse monoclonal anti-E-cadherin clone M168, Abnova, Cat# MAB1388, RRID:AB_1671631. Our data show that this antibody reacts

as expected based on morphology and western blots. http://www.abnova.com/products/products_detail.asp?catalog_id=MAB1388
Mouse monoclonal anti-GFP, clone GFP-G1, Developmental Studies Hybridoma Bank (DSHB), RRID: AB_2619561. Our data show that this antibody reacts with GFP-expressing cells but not with cells that lack GFP. https://dshb.biology.uiowa.edu/GFP-G1 (no catalog number available, please refer to website link provided)
Mouse monoclonal anti-GFP, Developmental Studies Hybridoma Bank (DSHB), Cat# DSHB-GFP-12E6 clone, RRID: AB_2619561, Our data show that this antibody reacts with GFP-expressing cells but not with cells that lack GFP. https://dshb.biology.uiowa.edu/ DSHB-GFP-12E6
Mouse monoclonal anti-occludin Invitrogen Cat. 33-1500, RRID:AB_2533101. We have found that this antibody does not react with occludin KO cells or mouse tissues. https://www.thermofisher.com/antibody/product/Occludin-Antibody-clone-OC-3F10-Monoclonal/33-1500
Mouse monoclonal anti-ZO1 Invitrogen Cat. 33-9100,clone 1A12. RRID:AB_2533147. We have found that this antibody does not react with ZO-1 KO cells or mouse tissues. https://www.thermofisher.com/antibody/product/ZO-1-Antibody-clone-ZO1-1A12-Monoclonal/33-9100
Mouse anti-caveolin-1 Invitrogen Cat. 03-6000, RRID:AB_2532932. KO verified by the manufacturer. https://www.thermofisher.com/antibody/product/caveolin-1-Antibody-clone-Z034-Monoclonal/03-6000
Mouse monoclonal anti-clathrin heavy chain Invitrogen Cat. MA1-065 X22, RRID: AB_2083179. Cell line knockdown verified. https://www.thermofisher.com/antibody/product/Clathrin-Heavy-Chain-Antibody-clone-X22-Monoclonal/MA1-065
Rabbit anti-claudin-3 Invitrogen Cat. 34-1700, RRID: AB_2533158, cell treatment verified. https://www.thermofisher.com/antibody/product/Claudin-3-Antibody-Polyclonal/34-1700
Rabbit anti-claudin-7 Invitrogen Cat. 34-9100, RRID:AB_2533190. cell treatment verified. https://www.thermofisher.com/antibody/product/Claudin-7-Antibody-Polyclonal/34-9100
Rabbit anti-EEA-1 Abcam ab2900, AB_2262056. KO validated by manufacturer. https://www.abcam.com/eea1-antibody-early-endosome-marker-ab2900.html
Rabbit anti-Rab5 Cell Signaling Technology, Cat# 3547, clone C8B1, RRID:AB_2300649. https://www.cellsignal.com/products/primary-antibodies/rab5-c8b1-rabbit-mab/3547
Rabbit anti-Rab7 Cell Signaling Technology, Cat# 9367, clone D95F2, RRID:AB_1904103. https://www.cellsignal.com/products/primary-antibodies/rab7-d95f2-xp-rabbit-mab/9367
Rat monoclonal anti-occludin J. Turner Clone. 6B8A3, RRID: AB_2819194. Cell and animal knockout verified by J Turner lab.
Rat monoclonal anti-ZO1 (originally sourced from the Daniel Goodenough lab, Harvard Medical School) Clone. R40.76, RRID:AB_2783859 Cell and knockout verified by J Turner lab. no catalog number available. Sold commercially by Millipore-Sigma (Cat # MABT11).
SECONDARY ANTIBODIES:
Abberior STAR RED goat anti-mouse IgG Abberior Cat #STRED-1001-20UG, https://abberior.shop/abberior-STAR-RED-goat-anti-mouse-IgG-500-ll-1-mg-ml.
IRDye 800CW goat anti-rabbit IgG LI-COR Biosciences Cat. 925-32211, RRID: AB_2651127. https://www.licor.com/bio/reagents/irdye-800cw-goat-anti-rabbit-igg-secondary-antibody.
IRDye 680LT goat anti-rabbit IgG LI-COR Biosciences Cat. 926-68021, RRID: AB_ 10706309. https://www.licor.com/bio/reagents/irdye-680lt-goat-anti-rabbit-igg-secondary-antibody.
IRDye 680RD-goat anti-mouse IgG LI-COR Biosciences Cat. 926-68070, RRID: AB_2651128. https://www.licor.com/bio/reagents/irdye-680rd-goat-anti-mouse-igg-secondary-antibody.
IRDye 800CW-goat anti-mouse IgG LI-COR Biosciences Cat. 926-32210, RRID AB_621842. https://www.licor.com/bio/reagents/irdye-800cw-goat-anti-mouse-igg-secondary-antibody.
Alexa 647 donkey anti-rat IgG highly cross-adsorbed F(ab')2 fragments Jackson ImmunoResearch Cat. 712-606-153; RRID:AB_2340696, 35 citations. https://www.jacksonimmuno.com/catalog/products/712-606-153.
Alexa 647-donkey anti-mouse IgG highly cross-adsorbed F(ab')2 fragments Jackson ImmunoResearch Cat. 715-606-151; RRID:AB_2340866, 39 citations. https://www.jacksonimmuno.com/catalog/products/715-606-151.
Alexa 647-donkey anti-rabbit IgG highly cross-adsorbed F(ab')2 fragments Jackson ImmunoResearch Cat. 711-606-152; RRID:AB_2340625, 82 citations. https://www.jacksonimmuno.com/catalog/products/711-606-152.

# Eukaryotic cell lines

Policy information about cell lines

| | |
|---|---|
| Cell line source(s) | U2OS osteosarcoma cell line were from ATCC. MDCK I cells were a gift from Dr. Enrique Rodriguez-Boulan (Cornell, New York, NY). MDCK II cells were a gift from Dr. James Anderson (NHLBI, Bethesda, MD). All other cell lines used here are derived from the parental lines and were developed in the corresponding author's laboratory. |
| Authentication | The CRISPR-mediated claudin-4 knockout cells were authenticated in detail by sequencing, immunostaining and western blots (Fig 1 and supplementary data Fig 1). Subsequent lines that were developed from these knockouts , as well as lines developed from U2OS cells were also subject to immunostaining and western blotting for verification of expression of the desired proteins (claudin-2 and claudin-4) using claudin-2 and claudin-4 cytoplasmic domain specific antibodies. |
| Mycoplasma contamination | All cell lines used in this study were routinely tested and found to be negative for mycoplasma using a PCR based detection kit (VenorGem- Sigma Aldrich) |
| Commonly misidentified lines (See ICLAC register) | no commonly misidentified cell lines were used in this paper. |
